# Supplementary material for: Investigating the characteristics and needs of frequently admitting hospital patients: a cross-sectional study in the UK
Source: BMJ Open. 2020 Sep 2;10(9):e035522. doi: 10.1136/bmjopen-2019-035522 (PMC7470484; doi:10.1136/bmjopen-2019-035522)
Supplement: Supplementary data [file bmjopen-2019-035522supp001.pdf]

Section A: YOUR ADMISSION EXPERIENCE

This part is about your experience while you were being ***admitted and treated*** *at Croydon University Hospital.*

1) What was the reason for your last admission at Croydon University Hospital?

2) Who decided that you need to go to A&E?

- ☐ Self
- ☐ Family
- ☐ GP
- ☐ Ambulance paramedic
- ☐ I don't know
- ☐ Other *(please specify)*

3) Did you try to seek help from any of the following before attending A&E? ***(please select all that apply)***

- ☐ None. I went directly to A&E
- ☐ Calling 111
- ☐ Contacting GP
- ☐ Visiting Walk-in centre
- ☐ Self-Care from the Pharmacy
- ☐ Community nurses
- ☐ HOT Clinics
- ☐ Other *(please specify)*

4) What day/time did you arrive to A&E?*(please select one box only)*

Day/Time	6am-12noon	12noon - 6pm	6pm-12midnight	12midnight - 6am
Weekday (Mon-Fri)	☐	☐	☐	☐
Weekend (Sat-Sun)	☐	☐	☐	☐

5) Following your arrival at A&E, how long did you wait before you were examined by a doctor?

- ☐ 1-2 Hours
- ☐ 2-3 Hours
- ☐ 3-4 Hours
- ☐ > 4 Hours *(please specify)*

6) Following examination by a doctor, how long did you wait before you were admitted to a bed in the ward?

- ☐ 1-2 Hours
- ☐ 2-3 Hours
- ☐ 3-4 Hours
- ☐ > 4 Hours *(please specify)*

7) To what extent do you agree/disagree that the following were explained to you in a way you could clearly understand

i The reasons for your admission

- ☐ Strongly Disagree
- ☐ Disagree
- ☐ Neutral
- ☐ Agree
- ☐ Strongly Agree

ii The decisions regarding your care, treatment, and/or procedure

- ☐ Strongly Disagree
- ☐ Disagree
- ☐ Neutral
- ☐ Agree
- ☐ Strongly Agree

8) Were you consulted regarding the decisions about your care, treatment, and/or procedure on admission?

- ☐ Yes
- ☐ No
- ☐ Can't Remember

9) In the future, would you like to be involved in decisions about your care, treatment, or procedure?

- ☐ Yes
- ☐ No
- ☐ Can't Remember

10) What could have improved your experience while being admitted to the hospital?

PROPRIETARY RIGHTS STATEMENT
This document contains information, which is proprietary to the OPTIMAL Consortium. Neither this document nor the information contained herein shall be used, duplicated or communicated by any means to any third party, in whole or in parts, except with prior written consent of the OPTIMAL consortium.

Section B: YOUR DISCHARGE EXPERIENCE

The following part is about your experience while you were being ***discharged from the hospital****.*

1) Were you informed 24 hours in advance about the discharge decision?

- ☐ Yes
- ☐ No
- ☐ Can't Remember

2) What day/time were you discharged from the hospital? *(Please tick one box only)*

Day/Time	6am-12noon	12noon-6pm	6pm-12midnight	12midnight-6am
Weekday (Mon-Fri)	☐	☐	☐	☐
Weekend (Sat-Sun)	☐	☐	☐	☐

3) Who provided you with the information related to your discharge? *(please select all that apply)*

- ☐ Doctor
- ☐ Nurse
- ☐ Pharmacist
- ☐ No one
- ☐ Other (please specify)

4) To what extent do you agree/disagree with the following:

i The decisions regarding my discharge were explained to me in a way I could clearly understand

- ☐ Strongly Disagree
- ☐ Disagree
- ☐ Neutral
- ☐ Agree
- ☐ Strongly Agree

ii I was fully consulted with the decision of being discharged from the hospital

- ☐ Strongly Disagree
- ☐ Disagree
- ☐ Neutral
- ☐ Agree
- ☐ Strongly Agree

iii Staff took my preferences into account in deciding how my health care will be managed when I will leave the hospital

- ☐ Strongly Disagree
- ☐ Disagree
- ☐ Neutral
- ☐ Agree
- ☐ Strongly Agree

5) Was there any change in your medicines during your last hospital admission?

- ☐ Yes
- ☐ No
- ☐ Can't Remember

6) Were you provided with any counselling about your medication(s)?

- ☐ Yes (go to 7)
- ☐ No (go to 12)
- ☐ Can't Remember

7) Who provided you with the information related to your medication(s) at discharge?
(please select all that apply)

- ☐ Doctor
- ☐ Nurse
- ☐ Pharmacist
- ☐ Other please specify

8) How was this information given to you?

- ☐ Verbally
- ☐ Written
- ☐ Verbally & Written

9) What resources were you given to help you take your medicine(s)? *(please select all that apply)*

- ☐ Patient information leaflet in box
- ☐ Medication reminder card
- ☐ Medication record book
- ☐ Poster or brochure
- ☐ None
- ☐ Other (please specify)

10) To what extent do you agree/disagree with the following:

i The information about my medication(s) were given/explained to me in a way I could clearly understand

- ☐ Strongly Disagree
- ☐ Disagree
- ☐ Neutral
- ☐ Agree
- ☐ Strongly Agree

ii I would like to have more information regarding my medicines

- ☐ Strongly Disagree
- ☐ Disagree
- ☐ Neutral
- ☐ Agree
- ☐ Strongly Agree

Kayyali R, *et al. BMJ Open* 2020; 10:e035522. doi: 10.1136/bmjopen-2019-035522

11) Please rate your satisfaction with regards to the following information about your medication, if provided during the counselling session:

Please tick one box for each row	Very Unsatisfied	Unsatisfied	Neutral	Satisfied	Very Satisfied	Not provided
Purpose of your medicine(s)	☐	☐	☐	☐	☐	☐
How to take/use the medicine(s)	☐	☐	☐	☐	☐	☐
Important side effects	☐	☐	☐	☐	☐	☐
Actions to take if you get any important side effects.	☐	☐	☐	☐	☐	☐
Lifestyle changes associated with taking your medicine(s)	☐	☐	☐	☐	☐	☐

12) **Community pharmacies are offering a New Medicines Service (NMS) which is an open conversation between you and the pharmacist to discuss any concerns you may have about your new medicine(s) - for example side effects**

i Are you aware of this service?
- Yes
- No

ii Were you offered/referred to this service upon discharge?
- Yes
- No
- Not Sure

iii Would you have been interested to be referred to this service?
- Yes
- No
- Not Sure

13) **Community pharmacies are offering a Medicines Use Review (MUR) which is an open conversation between you and the pharmacist to discuss your medications after you were discharged from the hospital or periodically.**

i Are you aware of this service?
- Yes
- No

ii Were you offered/referred to this service upon discharge?
- Yes
- No
- Not Sure

iii Would you have been interested to be referred to this service?
- Yes
- No
- Not Sure

14) **When discharged from the hospital, were you given a written copy of your care plan?**

☐ Yes
☐ No *(go to 15)*

i If yes, did you understand what was in this care plan?

☐ Yes
☐ No

15) **Were you referred to a post-discharge service**? *(e.g. hospital avoidance team, hot clinics, telehealth, community services nurse, social care)*

☐ Yes
☐ No *(go to 16)*

i If yes, please specify

ii Were you offered a choice to select those services?

☐ Yes
☐ No

iii Have you joined any of these services yet?

☐ Yes
☐ No

16) **Were you told about signs/signals of worsening or decline of your health to watch out for?**

☐ Yes
☐ No *(go to 17)*

i If yes, were you given details of who to contact if this happened?

☐ Yes
☐ No

17) **Were you given contacts for out-of-hours help?**

☐ Yes
☐ No

Kayyali R, *et al. BMJ Open* 2020; 10:e035522. doi: 10.1136/bmjopen-2019-035522

18) **Were you given information about local support groups?** *(E.g. Diabetes UK, Age UK, Cardiac Support Group, Breathe Easy groups, etc…)*

- ☐ Yes
- ☐ No

19) What could have improved your experience while being discharged from the hospital?

Section C: YOUR POST-DISCHARGE EXPERIENCE

*This part is about your experience since your **last hospital discharge***

1 How confident are you in managing your health?

- ☐ Not at all confident
- ☐ Not confident
- ☐ Neither
- ☐ Confident
- ☐ Completely Confident

i Why is that?

2 How worried have you been about being readmitted to the hospital?

- ☐ Very Worried
- ☐ Worried
- ☐ Neither
- ☐ Not Worried
- ☐ Not worried at all

i Why is that?

3 **Please rate your satisfaction with regards to the following**

Please tick one box for each row	Very Unsatisfied	Unsatisfied	Neutral	Satisfied	Very Satisfied	Not Applicable
The available support to manage your health after you were discharged from the hospital	☐	☐	☐	☐	☐	☐
Social care support after being discharged from the hospital *(e.g. help in your home, community support, etc…)*	☐	☐	☐	☐	☐	☐
Occupational needs support *(e.g. walking aids)*	☐	☐	☐	☐	☐	☐

4 When discharged from the hospital, were you given any follow up appointments?

- Yes
- No *(please go to 5)*
- Not Sure *(please go to 5)*

i If yes, who was the follow up appointment with? *(please select all that apply)*

- GP
- Nurse
- Hospital Outpatient
- Pharmacist
- Other (please specify)

ii Were you able to attend all these appointments?

- Yes
- No

iii If no, why is that? (Optional)

iv To what extent do you agree/disagree with the following:

(a) I clearly understand my post-discharge care plan/appointments

- Strongly Disagree
- Disagree
- Neutral
- Agree
- Strongly Agree

(b) I could easily keep track/record of my appointments

- Strongly Disagree
- Disagree
- Neutral
- Agree
- Strongly Agree

(c) I prefer to have my appointments available for me electronically (e.g. smart phone calendar)

- Strongly Disagree
- Disagree
- Neutral
- Agree
- Strongly Agree

5 Have you received any follow up from the hospital?

- Yes
- No *(please go to 6)*

Kayyali R, *et al. BMJ Open* 2020; 10:e035522. doi: 10.1136/bmjopen-2019-035522

- Don't remember *(please go to 6)*

i If yes, how? *(please select all that apply)*
- By phone call
- By letter
- By email
- By text message (SMS)
- Other (please specify)

ii When?
- 1 week after discharge
- 2 week after discharge
- 3 week after discharge
- 4 week after discharge
- Other (please specify)

6 Have you been contacted by any other post-discharge service team? (e.g. hospital avoidance team, hot clinics, telehealth, community services nurse, social care)
- Yes
- No *(please go to 7)*
- Don't remember *(please go to 7)*

i If yes, who was it? (please specify)

ii How? *(please select all that apply)*
- By phone call
- By letter
- By email
- By text message (SMS)
- Other *(please specify)*

iii When?
- 1 week after discharge
- 2 week after discharge
- 3 week after discharge
- 4 week after discharge
- Other *(please specify)*

7 During the first 30 days after you were discharged from the hospital:

i Were you contacted by any of the following *(please select all that apply)*
- GP
- Hospital
- Pharmacy Team
- Post-discharge Service
- None
- Other *(please specify)*

ii Did you contact any of the following *(please select all that apply)*
- GP
- Hospital
- Pharmacy Team
- Post-discharge Service
- None
- Other *(please specify)*

8 How confident are you regarding the management of the following:

Please tick one box for each row	Not at all confident	Not confident	Neither	Confident	Completely Confident	Not applicable
Your supply of medicines	☐	☐	☐	☐	☐	☐
Your social care issues	☐	☐	☐	☐	☐	☐
Your healthcare issues	☐	☐	☐	☐	☐	☐

Kayyali R, *et al. BMJ Open* 2020; 10:e035522. doi: 10.1136/bmjopen-2019-035522

Section D: SOME INFORMATION ABOUT YOU

1) How many times were you admitted to the hospital

in the last 12 months?	
in the last 30 days?	

1) **In what year were you born?**

2) What is the first part of your postcode? (E.g. if your post code is CR7 7YE, please write CR7)

3) What is your gender?

□ Male □Female

4) How would you describe your ethnicity?

- White
- Black
- Chinese
- Mixed
- Asian
- Other
- **Prefer** not to say

5) What is/are the main language(s) spoken at your home?

6) How often do you need to have someone help you when you read instructions, pamphlets, or other written material from your doctor or pharmacy

- Never
- Rarely
- Sometimes
- Often
- Always

7) Do you suffer from any medical conditions? If yes please specify

8) How often do you have a drink containing alcohol?

- Never *(please skip to 11)*
- Monthly or less
- 2 to 4 times a MONTH
- 2 to 3 times a WEEK
- 4 or more times a week

9) How many drinks of alcohol do you drink on a typical day when you are drinking?

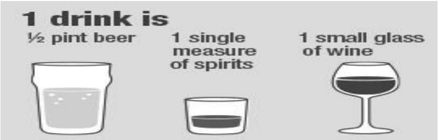

- 1 or 2 drinks
- 3 or 4 drinks
- 5 or 6 drinks
- 7 or 8 or 9 drinks
- 10 **or** more drinks

10) How often have you had 6 or more units on a single occasion in the last year?

- Never
- Less than monthly
- Monthly
- Weekly
- **Daily** or almost daily

11) Have you ever smoked a cigarette, a cigar or a pipe?

- Yes
- No *(please go to 12)*

i Yes, and I am currently a smoker

- How frequently do you smoke cigarettes?
  - Regularly
  - Occasionally
- (b) How many cigarettes do you smoke per day?
  - 10 or Less
  - 11-20
  - 21-30
  - 31 or More

ii Yes, but I am an ex-smoker

- How frequently did you smoke cigarettes?
  - Regularly
  - Occasionally
- (b) About how many cigarettes did you smoke in a day?
  - ☐ 10 or Less
  - ☐ 11-20
  - ☐ 21-30
  - ☐ 31 or More
- (c) For approximately how many years did you smoke cigarettes regularly?
- (d) How long ago did you stop smoking cigarettes?

iii Are you currently using e-cigarettes?

- ☐ Yes
- ☐ No

12) How many regular medicines are you currently taking?

13) How often do you miss doses of your medicine(s), cut down or stop taking them?

- ☐ Frequently (more than once a week)
- ☐ Occasionally (once a week)
- ☐ Rarely (once a month)
- ☐ Very rarely (once every 6 month)
- ☐ Never

14) Do you experience any of the following problems with your medicines which make it difficult for you to take your medicine(s)? (please select all that apply)

- ☐ Forget
- ☐ Cost
- ☐ Difficulty understanding the instructions
- ☐ Difficulty reading labels
- ☐ Difficulty opening containers

- ☐ Difficulty in administration *(e.g. difficulty in using Inhalers, swallowing tablets, or injection site, etc…)*
- ☐ Other *(please specify)*

15) Usually, how are your medicines supplied to you?

☐ Original or labelled box/bottle	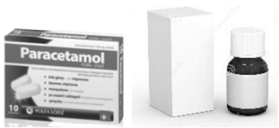
☐ Blister pack	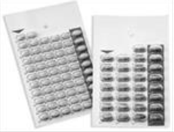
☐ Medicine Dosettes	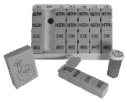

16) If known, please list your current medicines names

17) Who lives with you at home?

- ☐ I live alone
- ☐ I live with a family member
- ☐ I live with a friend
- ☐ I live in a care home
- ☐ Other please specify

18) Do you have someone who can take care of you?

- ☐ Yes
- ☐ No

i. Who provides you with home care?

- ☐ Family member
- ☐ Friend
- ☐ Carer
- ☐ Other please specify

ii. How many days a week are he/she/they available to you?

- ☐ < 1day
- ☐ 1-2 days
- ☐ 3-4 days
- ☐ 5-7 days

iii. When are they available to you?

- ☐ Weekdays only
- ☐ Weekend only
- ☐ Both
