# Supplementary material for: Investigating the characteristics and needs of frequently admitting hospital patients: a cross-sectional study in the UK
Source: BMJ Open. 2020 Sep 2;10(9):e035522. doi: 10.1136/bmjopen-2019-035522 (PMC7470484; doi:10.1136/bmjopen-2019-035522)
Supplement: Author's manuscript [file bmjopen-2019-035522.draft_revisions.pdf]

BMJ Open

BMJ Open is committed to open peer review. As part of this commitment we make the peer review history of every article we publish publicly available.

When an article is published we post the peer reviewers' comments and the authors' responses online. We also post the versions of the paper that were used during peer review. These are the versions that the peer review comments apply to.

The versions of the paper that follow are the versions that were submitted during the peer review process. They are not the versions of record or the final published versions. They should not be cited or distributed as the published version of this manuscript.

BMJ Open is an open access journal and the full, final, typeset and author-corrected version of record of the manuscript is available on our site with no access controls, subscription charges or pay-per-view fees (http://bmjopen.bmj.com).

If you have any questions on BMJ Open's open peer review process please email info.bmjopen@bmj.com

BMJ Open

Investigating the Characteristics and Needs of Frequently Readmitting Hospital Patients - A Cross-Sectional Study

Journal:	*BMJ Open*
Manuscript ID	bmjopen-2019-035522
Article Type:	Original research
Date Submitted by the Author:	15-Nov-2019
Complete List of Authors:	Kayyali, Reem; Kingston University Faculty of Science Engineering and Computing, Pharmacy<br>Funnell, Gill; Kingston University, Kingston University Faculty of Science Engineering and Computing<br>Odeh, Bassel; Kingston University Faculty of Science Engineering and Computing, Pharmacy<br>Sharma, Anuj; exus, Exus Innovations Tower 42, 25, Old Broad St, London EC2N 1PB<br>Katsaros, Yannis; exus, Exus Innovations, Tower 42, 25, Old Broad St, London EC2N 1PB<br>Nabhani-Gebara, Shereen; Kingston University Faculty of Science Engineering and Computing, Pharmacy<br>Pierscionek, Barbara ; Nottingham Trent University<br>Chang, John; Croydon University Hospital, Chest Clinic and Research and Development
Keywords:	Quality in health care < HEALTH SERVICES ADMINISTRATION & MANAGEMENT, HEALTH SERVICES ADMINISTRATION & MANAGEMENT, Organisation of health services < HEALTH SERVICES ADMINISTRATION & MANAGEMENT

SCHOLARONE™
Manuscripts

BMJ

I, the Submitting Author has the right to grant and does grant on behalf of all authors of the Work (as defined in the below author licence), an exclusive licence and/or a non-exclusive licence for contributions from authors who are: i) UK Crown employees; ii) where BMJ has agreed a CC-BY licence shall apply, and/or iii) in accordance with the terms applicable for US Federal Government officers or employees acting as part of their official duties; on a worldwide, perpetual, irrevocable, royalty-free basis to BMJ Publishing Group Ltd ("BMJ") its licensees and where the relevant Journal is co-owned by BMJ to the co-owners of the Journal, to publish the Work in this journal and any other BMJ products and to exploit all rights, as set out in our licence.

The Submitting Author accepts and understands that any supply made under these terms is made by BMJ to the Submitting Author unless you are acting as an employee on behalf of your employer or a postgraduate student of an affiliated institution which is paying any applicable article publishing charge ("APC") for Open Access articles. Where the Submitting Author wishes to make the Work available on an Open Access basis (and intends to pay the relevant APC), the terms of reuse of such Open Access shall be governed by a Creative Commons licence – details of these licences and which Creative Commons licence will apply to this Work are set out in our licence referred to above.

Other than as permitted in any relevant BMJ Author's Self Archiving Policies, I confirm this Work has not been accepted for publication elsewhere, is not being considered for publication elsewhere and does not duplicate material already published. I confirm all authors consent to publication of this Work and authorise the granting of this licence.

For peer review only

Investigating the Characteristics and Needs of Frequently Readmitting Hospital Patients- A Cross-Sectional Study

Author and Co-authors:

Reem Kayyali PhD, Gill Funnell MPharm, Bassel Odeh PhD, Anuj Sharma MSc, Yannis Katsaros PhD, Shereen Nabhani-Gebara PharmD, Barbara Pierscionek PhD, John Chang FRCP

Corresponding author:

Prof Reem Kayyali

School of Life Sciences, Pharmacy and Chemistry, Kingston University,

Penrhyn Road, Kingston upon Thames

Surrey, KT1 2EE, United Kingdom

Email: R.Kayyali@kingston.ac.uk

Telephone: +44 (0)20 8417 2651 (Internal: 62561)

Co-author details:

Gill Funnell, Bassel Odeh, Shereen Nabhani- Gebara

Affiliated to

School of Life Sciences, Pharmacy and Chemistry,

Kingston University, Penrhyn Road, Kingston upon Thames

Surrey, KT1 2EE, United Kingdom

Yannis Katsaros, Anuj Sharma

Affiliated to

Exus Innovations Tower 42, 25 Old Broad Street, London EC2N 1PB, United Kingdom

John Chang

Affiliated to

Department of Research and Development, Croydon University Hospital, London Rd CR7 7YE United Kingdom

London Road CR7 7YE United Kingdom

Barbara Pierscionek

Affiliated to Department of Science and Technology, Nottingham Trent University, Clifton Campus, Clifton Lane, NG11 8NS United Kingdom

Keywords: Patient Readmission, Patient Discharge, Health Services for the Elderly, Integrated Patient Care

Word Count: 4424

For peer review only - http://bmjopen.bmj.com/site/about/guidelines.xhtml

ABSTRACT

OBJECTIVES: This study forms the user requirements phase of the OPTIMAL project, which, through a predictive model and supportive intervention, aims to decrease early hospital readmissions. This phase aims to investigate the needs and characteristics of patients who had been readmitted to hospital ≥2 in the past 12 months.

SETTING: This was a cross-sectional study involving recent patients from Croydon University Hospital (CUH), London, UK

PARTICIPANTS: A total of 347 patients responded to a postal questionnaire, a response rate of 12.7%. To meet the inclusion criteria, participants needed to be aged ≥ 18 and have been admitted ≥2 times in the previous 12 months (August 2014-July 2015) to CUH.

PRIMARY AND SECONDARY OUTCOMES: To profile patients identified as frequent re-admitters to assess gaps in care at discharge or post discharge. Additionally, to understand the patients' experience of admission, discharge and post discharge care.

RESULTS: The range of admissions in the past 12 months was 2-30, with a mean of 2.8. At discharge 72.4%, (n= 231/347) were not given a contact for out of hours help. Regression analysis identified patient factors that were significantly associated with increased admissions, which included age (p=0.009), being in receipt of care (p=0.006) and admission due to a fall (p=0.008), but not receiving polypharmacy. Post-discharge, nearly half of patients (47.5%, n=145/305) were concerned about being readmitted to the hospital. In the first 30 days after discharge, over half of patients (54.5% n=189/347) had no contact from a health care professional.

For peer review only

CONCLUSION: Considering that social care needs were more of a determinant of readmission risk than medical needs, rectifying the lack of integration, communication and the under-utilisation of existing patient services could prevent avoidable problems during the transition of care and help decrease the likelihood of hospital readmission.

STRENGTHS AND LIMITATIONS OF THIS STUDY

1. This study identified that existing support services may be underutilised by patients post-discharge, highlighting the need to increase local service knowledge and referrals.
2. The study is representative of the population around CUH and is limited by the memory of the respondents.
3. The study gave an insight into the patient journey from admission, discharge to post-discharge, providing a holistic picture of patients' experiences. To our knowledge this is the first study covering all three periods.
4. Not all patients fully completed the questionnaire, hence, statistical significance was not achieved for the whole questionnaire.
5. The study highlighted that patients with social care needs were more likely to be readmitted than those with complex medical needs.

Introduction

A desire to reduce the increasing cost of healthcare provision is an impetus for many countries to search for new ways to both increase efficiency and improve the quality of hospital care. Reducing the cost of early hospital readmissions is an objective with clear benefits for both providers and patients[1].

For peer review only

For peer review only - http://bmjopen.bmj.com/site/about/guidelines.xhtml

In the UK, readmissions were estimated to cost the NHS £2.4 billion in 2012-2013, which is 19% of the total emergency admission cost of £12.5 billion.[2] Since 2011, UK hospitals have been financially penalised for patient readmissions occurring within 30 days of discharge.[3]

The UK financial penalty was introduced in 2011 to discourage hospitals from attempting to free up beds by discharging patients before they were ready.[3]However, not all readmissions are due to sub-optimal patient care and many readmissions may be unavoidable and appropriate, for example where patients are chronically or terminally ill.[4][5] Two UK studies found around 60% of early readmissions were due to the same reason as the primary admission, suggesting that these could have been reduced by medication reviews, better discharge communication and a rapid response to preventable issues. [6][7]

Both polypharmacy and chronic conditions such as chronic obstructive pulmonary disease (COPD), cardiovascular disease (CVD) and diabetes have been found to be associated with high readmissions rates and increased needs following discharge.[8][9] Accurately identifying patients as high risk enables resources to be channelled specifically to these patients through supportive interventions, rather than providing for all patients, many of whom may not be at risk of readmission. Several predictive models have been developed in the UK such as PARR-30[10] and in Canada the LACE[11] with relatively good predictive accuracy.

Evaluating the effectiveness of interventions designed to prevent early readmissions is problematic due to the lack of robust studies with good methodologies.[9] Intervention types which have been studied, often in combination include: Extensive discharge planning, telephone calls, home visits, a 24- hour hot line and patient education.[9] The provision of follow up telephone calls is a common intervention, with variation in the number and length of calls and profession of caller. The most successful results included both pre and post-discharge interventions. [12]

Schemes for supporting patients with their medications in the community were introduced into community pharmacies in 2005. Medicine Use Reviews (MURs) and New Medicines Service (NMS)

For peer review only - http://bmjopen.bmj.com/site/about/guidelines.xhtml

can support patients with medication adherence as well as identifying interactions and other problems. The NMS is specifically targeted at patients with long term conditions such as COPD to support patients starting a new medication[13].

The need for successful management of the pre and post-discharge period is highlighted in the National Institute for health and Care Excellence (NICE) Guidelines[14], developed in 2015 to help with the transition of adult patients with social care needs from hospital to the community. These guidelines emphasise the importance of the transition of care being co-ordinated using good communication. All healthcare professionals (HCPs) involved with the care of the patient in hospital and the community, should be included in the communication loop, with all patients/carers being provided with a medication list and a care plan with a single HCP responsible for co-ordinating the discharge for both social and medical needs.

This paper reports on the first stage of the OPTIMAL project, funded by Innovate UK. The OPTIMAL project encompasses the development of a predictive risk model, together with a supportive post-discharge patient intervention with the aim of reducing early hospital readmission. Although the success of both predictive risk models and interventions to prevent hospital readmission have been developed and studied separately before, this is the first time, to our knowledge, that a predictive model and a preventative intervention have been integrated to support patients.

The aim of this study was to undertake a needs assessment to investigate any common characteristics of patients identified as frequent admitters (≥2 in the past 12 months) and understand their experiences of both the discharge process and the immediate post-discharge period, with any difficulties which could contribute to readmission. This will assist in the development of an appropriate post-discharge intervention for patients identified at high risk of readmission.

METHODS

For peer review only - http://bmjopen.bmj.com/site/about/guidelines.xhtml

A cross-sectional study was carried out at Croydon University Hospital (CUH). Patients were considered for inclusion in the study if they met the following criteria: ≥18 years, a home address on the CUH database, experienced ≥ 2 admissions to CUH in the past 12 months (August 2014- July 2015) and were discharged between > 30 days and < 12 months ago. Paediatric, oncology and maternity patients were excluded from the study. CUH research and development (R&D) department using patient records identified a total of 2732 patients who met these inclusion criteria. To provide a confidence level of 95% and a confidence interval of 5%, the sample size was calculated as 337 patients. As a low response rate may be expected from postal survey, all 2732 patients were invited to complete the postal questionnaire. An explanatory letter was sent with the questionnaire together with a pre-paid return envelope. The questionnaire was only made available in English and no reminders were sent.

Ethical approval was obtained from Kingston University Delegated Research Ethics Committee (Ref: 1415/035) and approved by the R&D department by CUH as a service evaluation.

A quantitative cross-sectional questionnaire survey was designed using a mixture of open and closed questions. The validated tools AUDIT-C (a brief alcohol screening tool used to identify alcohol dependency)[15] and a medical health literacy score[16] were incorporated together with other questions which investigated patient experience and knowledge of medication and discharge counselling. The questionnaire was in four sections: Firstly, demographic information, collecting personal information such as age, as well as medication list and current medical conditions. Secondly, understanding the patient's admission experience, the reason for the patient's attendance at A&E and satisfaction with the admission process. Thirdly, the patient's discharge experience, investigating patients' involvement in their discharge planning and the provision of medication counselling. Finally, understanding the patients' post-discharge experience, the discharge support received by patients, as well as patients' confidence in managing their health and coping at home post-discharge.

For peer review only - http://bmjopen.bmj.com/site/about/guidelines.xhtml

Pilot

After receiving ethics approval, a pilot study was conducted which involved asking 10 patients from the discharge lounge at CUH to complete the survey for validation. Minor changes were made to the questionnaire. To prevent any bias, the findings from the pilot were not included in the final results.

Patient and Public Involvement

The study was a follow up study from 50 patients at the Trust who indicated mixed experience in counselling and shared decision making during admission. As part of the funding, the researchers the researchers agreed to inform patients/public of the outcome of the study which was done in the public engagement forums within the Trust.

Data Analysis

The responses from the returned questionnaires were analysed using IBM SPSS ver. 23® through descriptive statistics and the Chi-squared test for independence, with a level of significance set at 5% ($p<0.05$). A comorbidity polypharmacy score (CPS) was calculated (defined as the total of the number of pre-trauma comorbidities and the number of pre-admission medications in trauma patients ≥45 years). Our modified calculation was performed for all patients ≥45 years, using the number of medications specified in the questionnaire, together with the number of existing complaints recorded. A three question Audit-C score [15]was calculated, with each question having a possible score of 0-4 and giving a total score in the range between 0-12. A score of ≥ 5 is considered positive, indicating a higher risk of alcohol consumption. A single question health literacy tool was utilised giving scores of 1-5, with scores >2 indicating some difficulty reading printed health material. The number of medications most associated with adverse drug reactions (ADR) resulting in hospital admission was also recorded for each patient[17].

A linear regression analysis was carried out on the data to help identify significant patient characteristics which may have contributed to a greater number of admissions in the previous 12

For peer review only - http://bmjopen.bmj.com/site/about/guidelines.xhtml

months. This was carried out by adding a dependent variable column “frequent_admitter” to the data which was then assigned 1 if a patient’s admissions in the previous year were >2 or 0 if ≤2. The independent variables included in the regression analysis were: admission reason, (ethnicity, condition complexity indicator (which was set if a patient described their existing situation as complex/complicated or reported ≥ 2 conditions), a care indicator (identified by patients who were in receipt of some home care), CPS, patient age, number of medications. Any rows where any of these variables was missing was dropped from the regression analysis, thus leaving 169 patients to be included in the analysis.

RESULTS

The questionnaires were sent to 2722 patients, 347 were completed and returned giving a response rate of 12.7%. Valid percentages are reported due to respondents not always fully completing the questionnaire.

The most common reasons given for the last admission were respiratory problems such as asthma and COPD (15.0%, n=52). Nearly 10% (n=33) of patients were admitted due to a fall. Nearly a third (n=107) of patients reported more than one condition or described their condition as complex (Table 1).

Table 1 *Demographics and Medical Conditions of responders*

**Parameter**	**Number of Patients**
**Age**	
Mean Age (years)	69.2 (Range 18-100) sd 18.2 n=334
Gender	
Male	46.0% n=155/337
Female	54.0% n=182/337
**Ethnicity**	
White	75.1% n=250/333
Black	10.2% n=34/333
Chinese	0.9% n=3/333
Mixed	2.7% n=9/333
Asian	7.5% n=25/333
Other	2.7% n=9/333
Prefer not to say	0.9%n=3
**Medical History**	

For peer review only

No. Of admissions in past 12 months<br>Range 2-30	Mean=2.8<br>Mode= 2
No. of admissions in last 30 days<br>Range=1-6	n=32
**Most Common Reasons For Last Admission n=347**	
Respiratory problems eg Asthma, Breathing difficulties, Chest Infection, COPD, pneumonia	15.0% n=52
Pain: Chest Paint<br>Other Pain	5.2% n=18<br>5.8% n=20
Fall	9.5% n=33
Infections other than Chest	8.1% n=28
Cardiac conditions	6.16% n=23
Other	38.0% n=132
Not specified	11.8% n=41
**Most Common Existing Medical Conditions n=347**	
Cardiac Conditions	17% n=59
Respiratory Conditions	15.0% n=52
High Blood Pressure	11.8% n=41
Diabetes	12.1% n=42
No existing condition specified	35.4% n=123
>1 Long term condition (LTC) or described as "complex"	29.1% n=101

Over a quarter (28.8%, n=99/344) of patients lived alone and less than 5% (4.4% n=15/344) lived in a care home. Not all patients had someone to care for them; 26.7% (n=88/330) reported that they had no available care. Only 13.1% (n=43/328) of patients currently smoked, which is less than the UK average of 19% [18]. However, 39.3 % (n=129/328) described themselves as ex-smokers. Nearly a third of patients had a limited health literacy score (29.8%, n=101/339) and over 15% (16.6%, 30/180) had a positive AUDIT-C score associated with a higher alcohol consumption risk.

Admission

Over half of patients were referred to A&E by an HCP (59.3%, n=204/344), with just over a third (34.9%, n=120/344) of patients reporting that a family member or they themselves made the decision. Although, two-thirds of patients (69%, n=234/339) were consulted regarding admission and care decisions, nearly all patients (93.1%, n=311/334) wanted to be more involved with these

For peer review only - http://bmjopen.bmj.com/site/about/guidelines.xhtml

decisions. The most frequently expressed comments about the admission experience concerned communication problems and the lack of provision of information (41.1%, n=35/85).

Regression Analysis

Four variables were found to be significant predictors of >2 admissions in the previous 12 months. These were admission for a fall (p=0.008), not identifying as having a complex condition or reporting <2 conditions (p=0.002), age (p=0.009) and being in receipt of care at home (p=0.006). Additionally, the overall regression is significant according to the F test (F=0.03).

Discharge

Nearly half of patients, (44.1%, n=146/331) were not informed of the discharge decision 24 hours in advance, including 43.4% (n=43/99) of those who lived alone.

Over half of patients, (56.0%, n=187/334) were discharged from the hospital on a weekday between 12 noon and 6pm. However, a quarter of patients (22.2%, n=74/334) were discharged between 6pm-6am and 17.6% (n=13/74) of them lived alone with an average age of 71.2 years.

Nearly three-quarters (70.1% n=234/334) of patients agreed that the decisions regarding the discharge procedure were clearly explained (Table 2). However, only a third of patients (37.9%, n=119 /314) were provided with information to enable them to detect signs of deteriorating health. Furthermore, nearly three quarters of patients, (72.4% n=231) were not provided with contacts for out-of-hours support. Less than a third of patients were referred to a post-discharge service and less than half of respondents reported joining this service (Table 2).

Table 2 *Patients' Discharge Experience*

Patient Discharge Experience	Number of Patients
Received discharge information from a doctor	54.3% n=188/346
Felt the decisions at discharge were clearly explained	70.1% n=234/334
Was fully consulted in the decision of being discharged	67.9% n=226/333
Received a written copy of care plan	46.8% n=146/312

For peer review only

Told about signs or signals to watch out indicating health was worsening	37.9% n=119/314
Told who to contact if health deteriorated	70.6%n=84/119
Not told who to contact for out of hours help	72.4% n=231/319
Referred to a post-discharge service	30.2% n=95/315
Patient joined the post-discharge service	48.4% n=46/95
Provided with details of local support groups	19.9% n=63

Patients were asked their opinion about their discharge procedure and the main concerns expressed were the poor provision of information and communication difficulties at all levels. Patients' concerns included the lack of communication between hospital staff and the patients / patients' families (48.6% n=35/72), including two elderly patients discharged without informing their families. One patient stated: "more co-ordination is needed between the pharmacy and wards." Patients were also concerned about long waiting times (36.1% n=26/72), with 42.3% (n=11/26) of the waiting times involving a delay in receiving medications.

Medications

Over half of patients had their medications changed whilst in hospital (51.6%, n=176/341), but over a quarter of these patients, 28.4% (n=50/176) did not receive any counselling. Three quarters of patients (77.1%, n=165/214) agreed that medication information was explained in a way they could understand. However, 40.7% (n=81/199) would have liked more information regarding their medications.

The average number of medications per patient was 6.4 with of a range of 0 to 29. Only 13 patients (4.0%, n=13/321) reported taking no medications. Nearly two thirds (65.7%, n=211) of patients were taking $\geq$ 5 medications. The most common prescribed medication classes are shown in table 3.

Table 3 *Most Common Medication Classes*

**Medication Class**	**Number of Patients n=236**
Proton Pump Inhibitors	45.3% n=107
Statins	44.5% n=105
Antiplatelet drug	35.6% n=84

For peer review only - http://bmjopen.bmj.com/site/about/guidelines.xhtml

ACEI/ARBs (Angiotensin Converting Enzyme Inhibitors/ Angiotensin Receptor Blockers)	33.9% n=80
Beta Blockers	33.931.4% n=80
Calcium Channel Blockers	27.5% n=65
Loop Diuretics	22.0.% n=52
Opiod analgesics (Including tramadol)	13.6% n=32
Oral Anti-Coagulants	14.4% n=34
Beta2 Agonists	14.8% n=35

Some of the medication combinations found are not routinely recommended, due to being identified as risky [19]. For example, 11.0% (n=25/236) of patients were taking the high-risk combination of two or more anti-platelet drugs or an antiplatelet drug together with the anti-coagulant warfarin. Also 4% (n=10/236) were taking the high-risk triple combination of an (Angiotensin Converting Enzyme Inhibitors/ Angiotensin Receptor Blockers) ACEI/ARB, an (Non-Steroidal Anti-Inflammatory Drug (NSAID) and a diuretic.

Over half of patients (55.9%, n=132/236) were prescribed two or more 2 medications that could put them at high risk of admission due to an ADR (Table 4).

Table 4 *High Risk Drugs*[17]

Number of High Risk Meds	Number of Patients n =236
>5	0.85% n=2
5	6.4% n=15
4	5.9% n=14
3	20.8% n=49
2	22.0% n=52
1	22.0% n=52
0	22.0% n=52

The average calculated CPS score was 7.5. Scores greater than 7 are associated with an increased risk of falls and length of hospital stay, complications, short term and one year mortality [20], over 40% of patients (n=135/313) had scores >7 and 20 patients were considered as severe or morbid with scores between 15 and 32.

Post-Discharge Experience

For peer review only - http://bmjopen.bmj.com/site/about/guidelines.xhtml

While 72.2% (n=244/338) of patients were confident in managing their own health, nearly half (47.5% n=145/305) had concerns about being readmitted to the hospital, with two patients feeling that their last admission was due to medicine errors that could have been avoided. Receiving medication counselling in hospital (57.3%, n=191/333) was significantly associated with patients feeling more confident in the management of their health care issues (p=0.013). Over 85% of patients (86.1% n=260/302), were confident in managing their supply of medicines, but were less confident in managing their social care issues (58.0%, n=119/205) and healthcare issues (65.9%, n=170/258).

Over half of patients (55.3% n=163/295) were very satisfied or satisfied with the available support post-discharge in managing their health needs. However, less than half of patients were satisfied (45.2% n=95/210) with the support for their social care needs.

During the crucial first 30 days post-discharge from hospital, over half (54.5%, n=189/347) of patients did not receive any contact from a hospital, GP, pharmacy, or other post-discharge service. Only 17.6%, (n=61/347) of patients reported being contacted by their GP. During this time, patients were also very reticent to contact an HCP themselves, with only 12.1% (n=42/347) of patients reporting initiating contact.

Just under a quarter of patients (24.2% n=84/347) were contacted by other post-discharge services, of the 58 patients who specified a service, half were contacted by community or other nurse, but only three patients were referred to the respiratory HOT clinic, [21](a rapid access clinic to help patients with COPD avoid hospitalisation).

Community pharmacy support services were not well utilised post-discharge and only 5.3%, (n=17/319) of patients were referred to MUR, with 76.1% (n=242/318) of patients being unaware of MUR services. However, 55.0% (n=175/318) of patients were interested in receiving this service. Similarly, 84.0% (n=274/326) of patients were not referred to NMS, with 55.6% (n=179/322) of patients being interested in receiving this service.

For peer review only - http://bmjopen.bmj.com/site/about/guidelines.xhtml

DISCUSSION

This questionnaire-based study followed patients that had ≥ 2 hospital admissions/year living in the vicinity of CUH from admission, through discharge to post-discharge. Despite the low response rate, this is the first study that captures the complete patient journey from admission, discharge, through to post-discharge care. Furthermore, it identified characteristics of patients with high admission rates. A strength of this is the holistic nature of the reported data, which provides a comprehensive picture of these patients' experience of the support they were given, their physical health and medication when discharged from hospital. The data highlights a wide range of areas for improving patient support, including communication, utilisation and integration of services and medication counselling.

The study had several limitations: Firstly, it is representative of the population around CUH and is limited by the memory of the respondents. Secondly, not all patients fully completed the questionnaire, hence, statistical significance was not achieved for the whole questionnaire. Thirdly, as the questionnaire was only available in English, this limited the study to participants who had sufficient English, the black population was also under represented at 10.2% compared to the 2011 census figure of 20.2%. [22]Three quarters (75.1%) of patients described themselves ethnically as white, which is an over representation when compared to the Croydon borough 2011 census figure of 47.3%. [22]Fourthly, the regression analysis was constrained by the lack of a control group.

Regression analysis identified 4 patient characteristics associated with higher admission. It is interesting that two of these factors: falls and being in receipt of care, both require liaison with other services post-discharge to provide adequate support in the patient's home. Suffering from falls is a well-known cause of hospital readmission and corroborates with other studies [23][24]), but being in receipt of care is, as far as we are aware a novel, though not surprising reason for readmission. The only admission reason (see Table 1) that was significantly associated with increased admissions was

For peer review only - http://bmjopen.bmj.com/site/about/guidelines.xhtml

having a fall. In fact, falling was the second most common reason for admission as reported by nearly 10% of patients. Polypharmacy, higher CPS score and identifying one's condition as complex or having ≥2 existing conditions were not associated with higher numbers of admissions in this study. Medications may often be implicated in falls with an increased risk for patients found even with < 5 medications, however the medication class may be deemed to be more significant than the number. [25][26] Nevertheless, a higher CPS and taking ≥ 5 medications has been associated with an increase in falls by other studies. [27] Nearly 50% of patients had a CPS score ≥ 7 and 65% were taking 5 or more medications. It must be noted that in this study, medications and conditions were self-reported. However, these did not act as predictors of readmission, thus highlighting that social care needs are superseding medical needs in determining readmission risk. Additionally, it may be that patients who were more aware of their health situation were also conscious of their need for their medications and thus were more likely to adhere to their medications [28] and ask for support from HCP before hospitalisation was required. This may explain why those who identified their condition as less complex had a significantly higher rate of readmission.

Receipt of medication counselling was significantly associated with patient confidence in managing health (p=0.013). Medicine combinations were reported which could have been questioned, such as patients taking two anti-platelet drugs or an anti-platelet drug with warfarin, which can lead to an increase risk of bleeding.[29] Ten patients were taking the combination of a NSAID, ACEI/ARB together with a diuretic, this combination is associated with an increased risk of acute kidney injury.[29] Community pharmacists being the most accessible HCP, are well placed to identify medications which cause adverse events to patients and increase their risk of falls. Patients were not referred to, and had a lack of awareness of community pharmacy medicine information schemes - MUR or NMS. These schemes provide another opportunity to identify and investigate medication interactions. This was a missed opportunity for medication support post-discharge in the community. In fact , an initiative at CUH that piloted the provision of domiciliary MUR to

For peer review only - http://bmjopen.bmj.com/site/about/guidelines.xhtml

housebound 'high risk' patients by community pharmacists resulted in reported avoidance of hospitalisation.[30][31]

Although nearly three quarters of patients felt consulted in the decisions leading to their discharge, patients expressed dissatisfaction with the discharge process, with long waiting times, delays and poor communication reported as the most common complaints. These findings correlate with an AGE UK report [32] investigating older people's experience of hospital readmission. Delays in discharge and lack of information are upsetting and confusing, especially for some older patients. Patients should at least be provided with updates as to the progress of their discharge. Although this study is limited to the experiences of the population around Croydon, a study from Liverpool Hospital UK [33] reported similar percentages of patients (70%) who felt that discharge decisions were explained, with the long wait for discharge medications also having a negative influence on the discharge experience.

Nearly 50% of patients were worried about being readmitted to hospital and commented on finding the experience stressful and wanting to avoid readmission. Good communication and information sharing supports the transition from hospital and helps prevent readmission[14][34]. Contact information should be provided in case of a short-term crisis, which should be proactive rather than waiting for a more serious problem to arise. However, it was found that nearly 40% of patients were not provided with the signs of deterioration of their condition and nearly three quarters of patients were not provided with details of who to contact if this situation arose. This lack of information could result in patients returning to hospital. Additionally, patients' carers and families were not always informed of the discharge, making it hard for them to adequately support the patient at home.

Poor integration of services was found both within the hospital and between primary and secondary care providers. Patients with social care needs should be contacted by a GP or community nurse within 24-72 hours of discharge.[14] However, less than 20% of patients were contacted by their GP

For peer review only - http://bmjopen.bmj.com/site/about/guidelines.xhtml

within 30 days of discharge. A further 12.1% contacted a HCP themselves. Additionally, patients were not being referred to post-discharge services which could have supported them. Despite 20 patients reporting suffering from COPD and 13 of these patients reporting respiratory problems/exacerbation as the reason for admission, only 3 patients were referred to the respiratory HOT clinic at CUH[21] which provides an integrated team of multidisciplinary HCPs. Nearly a third of patients were dissatisfied with their social care, thus it is not surprising that those receiving care were more at risk of readmission. A lack of transition of care was reported, with a need for low level practical support during the first few days after discharge. This is a shared outcome with the AGE UK report. [32]

More integrated support such as that provided by Lewisham Integrated Medicines Optimisation Scheme (LIMOS) [35] can break through traditional boundaries of care, but as these authors note such links with services take time to build. With an increasing aging population with more multi-morbidities, the integration of service delivery across different clinical areas becomes more important to provide appropriate individual care, rather than the current disease-focused practice.[36] A move to a shared responsibility,[37] is required across multiple areas- social, voluntary and clinical, to provide the integrated personalised care patients need.

CONCLUSIONS

The study highlighted gaps in care during the patient discharge journey. Admission for a fall and receipt of care were significantly associated with higher admission rates. Additionally, it reports for the first time, that social care is a more important determinant of readmission in a predominantly older population than medical care. Before discharge, patients lacked medication counselling, information on symptoms of deteriorating health, or HCP to contact if this situation arose. An improvement in communications at all levels would benefit patients, ensuring patients are informed of delays and decisions. Additionally, patients' confidence in their care being well managed may be increased by demonstrating that communication channels are open between different HCPs. Post-

For peer review only - http://bmjopen.bmj.com/site/about/guidelines.xhtml

For peer review only

discharge, patients were lacking referrals to relevant services which could have supported them. The study highlighted that transitional care is fragmented between different services of primary, secondary and social care as well as the voluntary sector. This lack of integration is causing patients avoidable difficulties. Improvement could be made by increasing HCP awareness of the available services, both voluntary and statutory, in the local area and encouraging links. Integrating services would increase the utilisation of existing resources, such as community pharmacy medicine support schemes, hospital services, e.g. respiratory HOT clinics as well as voluntary services, with care pathways utilising all relevant services across each sector.

CONTRIBUTORSHIP STATEMENT RK was the principal investigator of the study. She was responsible for the design of the study. She also organised and co-ordinated all aspects of this research. GF worked alongside RK to draft the publication. BS contributed to data collection. The analysis of the results was carried out by RK, GF, BS, SE, YK, AS and JC.

COMPETING INTERESTS All authors have completed the ICMJE form for disclosure of potential conflicts of interest available from www.icmje.org/coi_disclosure.pdf and declare that there is nothing to disclose.

FUNDING: This research was carried out as part of the OPTIMAL project which has received funding from Innovate UK.

DATA SHARING STATEMENT No additional data is available

1. OECD Health Ministerial Meeting: Health System Priorities in the Aftermath of the Crisis [Internet]. 2010 [cited 2017 Feb 7]. p. 7–8. Available from: https://www.oecd.org/health/2010-ministerial/46098360.pdf
2. National Audit Office. Emergency admissions to hospital: managing the demand [Internet]. Vol. 739, Hc. 2013 [cited 2017 Feb 10]. p. 4. Available from: https://www.nao.org.uk/wp-content/uploads/2013/10/10288-001-Emergency-admissions.pdf
3. Department of Health. Payment by Results Guidance for 2011-12 [Internet]. 2011 [cited 2017 Feb 17]. Available from:

For peer review only - http://bmjopen.bmj.com/site/about/guidelines.xhtml

http://webarchive.nationalarchives.gov.uk/20130507170152/https://www.gov.uk/government/uploads/system/uploads/attachment_data/file/151911/dh_126157.pdf.pdf

4. Benbassat J, Taragin M. Hospital Readmissions as a Measure of Quality of Health Care. arch intern med. 2000;160:1074–81.

5. Lindquist LA, Baker DW. Understanding preventable hospital readmissions: Masqueraders, markers, and true causal factors. J Hosp Med. 2011;6(2):51–3.

6. Conroy SP et al. Understanding readmissions: An in-depth review of 50 patients readmitted back to an acute hospital within 30 days. Eur Geriatr Med [Internet]. 2013;4(1):25–7. Available from: http://dx.doi.org/10.1016/j.eurger.2012.02.007

7. Jakupaj A. Reducing readmission rate in the elderly population at a District General Hospital. Eur Geriatr Med [Internet]. 2014;5:S248–9. Available from: http://ovidsp.ovid.com/ovidweb.cgi?T=JS&PAGE=reference&D=emed12&NEWS=N&AN=71659663

8. Hurding S et al. Polypharmacy Guidance 2015 (NHS Scotland). 2015.

9. Jayakody A et al. Effectiveness of interventions utilising telephone follow up in reducing hospital readmission within 30 days for individuals with chronic disease: a systematic review. BMC Health Serv Res [Internet]. 2016;16(1):403. Available from: http://www.ncbi.nlm.nih.gov/pubmed/27538884%5Cnhttp://www.pubmedcentral.nih.gov/articlerender.fcgi?artid=PMC4990979

10. Billings J et al. Development of a predictive model to identify inpatients at risk of re-admission within 30 days of discharge (PARR-30). BMJ Open [Internet]. 2012;2(4):e001667–e001667. Available from: http://www.ncbi.nlm.nih.gov/pubmed/22885591%5Cnhttp://www.pubmedcentral.nih.gov/articlerender.fcgi?artid=PMC3425907

11. Spiva L et al. Validation of a Predictive Model to Identify Patients at High Risk for Hospital Readmission. J Heal Qual. 2015;00(0):1–7.

12. Sales VL et al. Utilization of Trained Volunteers Decreases 30-Day Readmissions for Heart Failure. J Card Fail [Internet]. 2014;20(5):377–377.e23. Available from: http://linkinghub.elsevier.com/retrieve/pii/S1071916414001432

13. NHS. Medicine Use Reviews [Internet]. [cited 2017 Apr 10]. Available from: http://www.nhsemployers.org/your-workforce/primary-care-contacts/community-pharmacy/medicines-use-reviews-murs

14. NICE NG27. NICE Transition between inpatient hospital settings and community or care home settings for adults with social care needs NG27 [Internet]. 2015 [cited 2017 Mar 5]. p. 1–29. Available from: https://www.nice.org.uk/guidance/ng27

15. Bush K et al. The AUDIT Alcohol Consumption Questions (AUDIT-C) An Effective Brief Screening Test for Problem Drinking. Arch Intern Med. 1998;158(16):1789–95.

16. Chew LD et al. Validation of screening questions for limited health literacy in a large VA outpatient population. J Gen Intern Med. 2008;23(5):561–6.

17. Pirmohamed M et al. Adverse drug reactions as cause of admission to hospital: prospective analysis of 18 820 patients. Bmj. 2004;329(7456):15–9.

18. Centre H and SCI. Statistics on Smoking - Key facts [Internet]. 2016 [cited 1917 Jan 9].

For peer review only - http://bmjopen.bmj.com/site/about/guidelines.xhtml

Available from: http://content.digital.nhs.uk/catalogue/PUB20781/stat-smok-eng-2016-app.pdf

19. All Wales Medicines Strategy Group. Polypharmacy: Guidance for Prescribing. July 2014. [Internet]. 2014 [cited 2017 Mar 7]. Available from: http://www.awmsg.org/docs/awmsg/medman/Polypharmacy - Guidance for Prescribing.pdf

20. Housley BC et al. Comorbidity-polypharmacy score predicts readmission in older trauma patients. J Surg Res [Internet]. 2015;199(1):237–43. Available from: http://dx.doi.org/10.1016/j.jss.2015.05.014

21. Croydon Health Care Trust. HOT Clinics [Internet]. 2012 [cited 2017 Apr 19]. Available from: https://www.croydonhealthservices.nhs.uk/new_page.htm

22. UK Office For National Statistics. 2011 UK Census Aggregate Data [Internet]. 2011 [cited 2017 Apr 10]. Available from: https://www.ons.gov.uk/census/2011census

23. Hughes LD, Witham MD. Causes and correlates of 30 day and 180 day readmission following discharge from a Medicine for the Elderly Rehabilitation unit. BMC Geriatr. 2018;18(1):1–10.

24. Hallgren J, Aslan AKD. Risk factors for hospital readmission among Swedish older adults. Eur Geriatr Med [Internet]. 2018;9(5):603–11. Available from: https://doi.org/10.1007/s41999-018-0101-z

25. Helgadóttir B et al. Medication and fall injury in the elderly population; Do individual demographics, health status and lifestyle matter? BMC Geriatr. 2014;14(1):1–8.

26. Fritsch MA, Shelton PS. Geriatric Polypharmacy: Pharmacist as Key Facilitator in Assessing for Falls Risk. Clin Geriatr Med. 2017;33(2):205–23.

27. Laflamme L et al. Type, number or both? A population-based matched case-control study on the risk of fall injuries among older people and number of medications beyond fall-inducing drugs. PLoS One. 2015;10(3):1–12.

28. Lee YM et al. Impact of health literacy on medication adherence in older people with chronic diseases. Collegian [Internet]. 2017;24(1):11–8. Available from: http://dx.doi.org/10.1016/j.colegn.2015.08.003

29. Lapi F et al. Concurrent use of diuretics, angiotensin converting enzyme inhibitors, and angiotensin receptor blockers with non-steroidal anti-inflammatory drugs and risk of acute kidney injury: nested case-control study. BMJ [Internet]. 2013;346(jan08_12):e8525. Available from: http://www.bmj.com/content/346/bmj.e8525

30. Jesson B, Williams V. Domiciliary Medicine Review Service in Croydon - Presentation [Internet]. Pharmacy Management National Forum - Forum Workshop. 2015 [cited 2017 Apr 7]. Available from: http://www.pharman.co.uk/imagelib/pdfs/Domiciliary_Medicine_Review_Service_in_Croydon_-_Barbara_Jesson_and_Victoria_Williams.pdf

31. Williams, V Jesson, B McCoig, A Beavon, N Rajah D. Domiciliary MURs: our experience in Croydon [Internet]. Pharmaceutical Journal. 2012 [cited 2017 Apr 9]. Available from: http://www.pharmaceutical-journal.com/opinion/correspondence/domiciliary-murs-our-experience-in-croydon/11109461.article

32. Lawrie M, Battye F. Older people's experience of emergency hospital readmission [Internet]. Age UK. 2012 [cited 2017 Mar 9]. Available from: http://www.ageuk.org.uk/Documents/EN-GB/For-

For peer review only - http://bmjopen.bmj.com/site/about/guidelines.xhtml

professionals/Research/Emergency_readmission_older_peoples_experiences.pdf?dtrk=true

33. Wright S et al. UK hospital patient discharge: the patient perspective. Eur J Hosp Pharm. 2017;(January).

34. Winfield A, Burns E. Let's all get home safely : a commentary on NICE and SCIE guidelines (NG27) transition between inpatient hospital settings and community or care home settings. Age Ageing. 2016;45(August):757–60.

35. Lai K et al. Lewisham integrated medicines optimisation service: delivering a system-wide coordinated care model to support patients in the management of medicines to retain independence in their own home. Eur J Hosp Pharm. 2015;22(2):98–101.

36. Stange KC, Ferrer RL. The Paradox of Primary Care (editorial). Ann Fam Med [Internet]. 2009;7(4):293–9. Available from: https://www.ncbi.nlm.nih.gov/pmc/articles/PMC2713149/pdf/0060293.pdf

37. Valentijn PP et al. Understanding integrated care: a comprehensive conceptual framework based on the integrative functions of primary care. Int J Integr Care. 2013;13(March).

For peer review only

STROBE 2007 (v4) Statement—Checklist of items that should be included in reports of *cross-sectional studies*

Section/Topic	Item #	Recommendation	Reported on page #
**Title and abstract**	1	(*a*) Indicate the study's design with a commonly used term in the title or the abstract	1
		(*b*) Provide in the abstract an informative and balanced summary of what was done and what was found	1, 2
**Introduction**			
Background/rationale	2	Explain the scientific background and rationale for the investigation being reported	3-5
Objectives	3	State specific objectives, including any prespecified hypotheses	5
**Methods**			
Study design	4	Present key elements of study design early in the paper	6
Setting	5	Describe the setting, locations, and relevant dates, including periods of recruitment, exposure, follow-up, and data collection	6
Participants	6	(*a*) Give the eligibility criteria, and the sources and methods of selection of participants	6
Variables	7	Clearly define all outcomes, exposures, predictors, potential confounders, and effect modifiers. Give diagnostic criteria, if applicable	
Data sources/ measurement	8*	For each variable of interest, give sources of data and details of methods of assessment (measurement). Describe comparability of assessment methods if there is more than one group	
Bias	9	Describe any efforts to address potential sources of bias	
Study size	10	Explain how the study size was arrived at	6
Quantitative variables	11	Explain how quantitative variables were handled in the analyses. If applicable, describe which groupings were chosen and why	7
Statistical methods	12	(*a*) Describe all statistical methods, including those used to control for confounding	7
		(*b*) Describe any methods used to examine subgroups and interactions	
		(*c*) Explain how missing data were addressed	
		(*d*) If applicable, describe analytical methods taking account of sampling strategy	
		(*e*) Describe any sensitivity analyses	
**Results**			

For peer review only

Participants	13*	(a) Report numbers of individuals at each stage of study—eg numbers potentially eligible, examined for eligibility, confirmed eligible, included in the study, completing follow-up, and analysed	8
		(b) Give reasons for non-participation at each stage	
		(c) Consider use of a flow diagram	
Descriptive data	14*	(a) Give characteristics of study participants (eg demographic, clinical, social) and information on exposures and potential confounders	8
		(b) Indicate number of participants with missing data for each variable of interest	
Outcome data	15*	Report numbers of outcome events or summary measures	8-13
Main results	16	(*a*) Give unadjusted estimates and, if applicable, confounder-adjusted estimates and their precision (eg, 95% confidence interval). Make clear which confounders were adjusted for and why they were included	
		(*b*) Report category boundaries when continuous variables were categorized	
		(*c*) If relevant, consider translating estimates of relative risk into absolute risk for a meaningful time period	
Other analyses	17	Report other analyses done—eg analyses of subgroups and interactions, and sensitivity analyses	
**Discussion**			
Key results	18	Summarise key results with reference to study objectives	14
Limitations	19	Discuss limitations of the study, taking into account sources of potential bias or imprecision. Discuss both direction and magnitude of any potential bias	14
Interpretation	20	Give a cautious overall interpretation of results considering objectives, limitations, multiplicity of analyses, results from similar studies, and other relevant evidence	16-17
Generalisability	21	Discuss the generalisability (external validity) of the study results	16-17
**Other information**			
Funding	22	Give the source of funding and the role of the funders for the present study and, if applicable, for the original study on which the present article is based	18

*Give information separately for cases and controls in case-control studies and, if applicable, for exposed and unexposed groups in cohort and cross-sectional studies.

Note: An Explanation and Elaboration article discusses each checklist item and gives methodological background and published examples of transparent reporting. The STROBE checklist is best used in conjunction with this article (freely available on the Web sites of PLoS Medicine at http://www.plosmedicine.org/, Annals of Internal Medicine at http://www.annals.org/, and Epidemiology at http://www.epidem.com/). Information on the STROBE Initiative is available at www.strobe-statement.org.

For peer review only

BMJ Open

Investigating the Characteristics and Needs of Frequently Admitting Hospital Patients - A Cross-Sectional Study

Journal:	*BMJ Open*
Manuscript ID	bmjopen-2019-035522.R1
Article Type:	Original research
Date Submitted by the Author:	01-Apr-2020
Complete List of Authors:	Kayyali, Reem; Kingston University Faculty of Science Engineering and Computing, Pharmacy<br>Funnell, Gill; Kingston University, Kingston University Faculty of Science Engineering and Computing<br>Odeh, Bassel; Kingston University Faculty of Science Engineering and Computing, Pharmacy<br>Sharma, Anuj; exus, Exus Innovations Tower 42, 25, Old Broad St, London EC2N 1PB<br>Katsaros, Yannis; exus, Exus Innovations, Tower 42, 25, Old Broad St, London EC2N 1PB<br>Nabhani-Gebara, Shereen; Kingston University Faculty of Science Engineering and Computing, Pharmacy<br>Pierscionek, Barbara; Staffordshire University, School of Life Sciences and Education<br>Wells, Joshua; Kingston University Faculty of Science Engineering and Computing, Pharmacy<br>Chang, John; Croydon University Hospital, Chest Clinic and Research and Development
**Primary Subject Heading**:	Health informatics
Secondary Subject Heading:	Health services research
Keywords:	Quality in health care < HEALTH SERVICES ADMINISTRATION & MANAGEMENT, HEALTH SERVICES ADMINISTRATION & MANAGEMENT, Organisation of health services < HEALTH SERVICES ADMINISTRATION & MANAGEMENT

SCHOLARONE™
Manuscripts

I, the Submitting Author has the right to grant and does grant on behalf of all authors of the Work (as defined in the below author licence), an exclusive licence and/or a non-exclusive licence for contributions from authors who are: i) UK Crown employees; ii) where BMJ has agreed a CC-BY licence shall apply, and/or iii) in accordance with the terms applicable for US Federal Government officers or employees acting as part of their official duties; on a worldwide, perpetual, irrevocable, royalty-free basis to BMJ Publishing Group Ltd ("BMJ") its licensees and where the relevant Journal is co-owned by BMJ to the co-owners of the Journal, to publish the Work in this journal and any other BMJ products and to exploit all rights, as set out in our licence.

The Submitting Author accepts and understands that any supply made under these terms is made by BMJ to the Submitting Author unless you are acting as an employee on behalf of your employer or a postgraduate student of an affiliated institution which is paying any applicable article publishing charge ("APC") for Open Access articles. Where the Submitting Author wishes to make the Work available on an Open Access basis (and intends to pay the relevant APC), the terms of reuse of such Open Access shall be governed by a Creative Commons licence – details of these licences and which Creative Commons licence will apply to this Work are set out in our licence referred to above.

Other than as permitted in any relevant BMJ Author's Self Archiving Policies, I confirm this Work has not been accepted for publication elsewhere, is not being considered for publication elsewhere and does not duplicate material already published. I confirm all authors consent to publication of this Work and authorise the granting of this licence.

For peer review only

Investigating the Characteristics and Needs of Frequently Admitting Hospital Patients- A Cross-Sectional Study

Author and Co-authors:

Reem Kayyali PhD, Gill Funnell MPharm, Bassel Odeh PhD, Anuj Sharma MSc, Yannis Katsaros PhD, Shereen Nabhani-Gebara PharmD, Barbara Pierscionek PhD, Joshua Wells MPharm, John Chang FRCP

Corresponding author:

Prof Reem Kayyali

School of Life Sciences, Pharmacy and Chemistry, Kingston University,

Penrhyn Road, Kingston upon Thames

Surrey, KT1 2EE, United Kingdom

Email: R.Kayyali@kingston.ac.uk

Telephone: +44 (0)20 8417 2651 (Internal: 62561)

Co-author details:

Gill Funnell, Bassel Odeh, Shereen Nabhani-Gebara, Joshua Wells

Affiliated to

School of Life Sciences, Pharmacy and Chemistry,

Kingston University, Penrhyn Road, Kingston upon Thames

Surrey, KT1 2EE, United Kingdom

Yannis Katsaros

Affiliated to

Medidata, 12 Hammersmith Grove, London W6 7AP, United Kingdom

Anuj Sharma

Affiliated to

Exus Innovations Tower 42, 25 Old Broad Street, London EC2N 1PB, United Kingdom

John Chang

Affiliated to

Department of Research and Development, Croydon University Hospital, London Rd CR7 7YE United Kingdom

London Road CR7 7YE United Kingdom

Barbara Pierscionek

School of Life Sciences and Education, Staffordshire University, College Road, ST4 2DE

Keywords: Patient Readmission, Patient Discharge, Health Services for the Elderly, Integrated Patient Care

For peer review only - http://bmjopen.bmj.com/site/about/guidelines.xhtml

Word Count: 4562

ABSTRACT

OBJECTIVES: This study forms the user requirements phase of the OPTIMAL project, which, through a predictive model and supportive intervention, aims to decrease early hospital readmissions. This phase aims to investigate the needs and characteristics of patients who had been admitted to hospital ≥2 times in the past 12 months.

SETTING: This was a cross-sectional study involving recent patients from Croydon University Hospital (CUH), London, UK

PARTICIPANTS: A total of 347 patients responded to a postal questionnaire, a response rate of 12.7%. To meet the inclusion criteria, participants needed to be aged ≥ 18 and have been admitted ≥2 times in the previous 12 months (August 2014-July 2015) to CUH.

PRIMARY AND SECONDARY OUTCOMES: To profile patients identified as frequent admitters to assess gaps in care at discharge or post-discharge. Additionally, to understand the patients' experience of admission, discharge and post-discharge care.

RESULTS: The range of admissions in the past 12 months was 2-30, with a mean of 2.8. At discharge 72.4%, (n= 231/347) were not given a contact for out of hours help. Regression analysis identified patient factors that were significantly associated with frequent admissions (>2 in 12 months), which included age (p=0.008), being in receipt of care (p=0.005) and admission due to a fall (p=0.01), but not receiving polypharmacy. Post-discharge, 41.8% (n=145/347) were concerned about being

For peer review only

readmitted to the hospital. In the first 30 days after discharge, over half of patients (54.5% n=189/347) had no contact from a health care professional.

CONCLUSION: Considering that social care needs were more of a determinant of admission risk than medical needs, rectifying the lack of integration, communication and the under-utilisation of existing patient services could prevent avoidable problems during the transition of care and help decrease the likelihood of hospital readmission.

STRENGTHS AND LIMITATIONS OF THIS STUDY

1. The study evaluated the patient journey from admission, discharge to post-discharge, providing a holistic picture of patients' experiences
2. The study successfully implemented a cross-sectional questionnaire across a diverse sample population using a postal survey method with no reminders sent
3. The target sample included all patients ≥18 years of age who experienced ≥2 admissions in the past 12 months at CUH
4. The study utilised linear regression analysis to identify significant contributing factors to patients being admitted >2 times in a 12 month period
5. The study is representative of patients admitted only to CUH and is limited by the memory of the respondents

Introduction

A desire to reduce the increasing cost of healthcare provision is an impetus for many countries to search for new ways to both increase efficiency and improve the quality of hospital care. Reducing

For peer review only - http://bmjopen.bmj.com/site/about/guidelines.xhtml

the cost of early hospital readmissions is an objective with clear benefits for both providers and patients.[1]

In the UK, readmissions were estimated to cost the NHS £2.4 billion in 2012-2013, which is 19% of the total emergency admission cost of £12.5 billion.[2] Since 2011, UK hospitals have been financially penalised for patient readmissions occurring within 30 days of discharge, which is considered as early readmission.[3]

The UK financial penalty was introduced in 2011 to discourage hospitals from attempting to free up beds by discharging patients before they were ready.[3] However, not all early readmissions are due to sub-optimal patient care and many readmissions may be unavoidable and appropriate, for example where patients are chronically or terminally ill.[4][5] Two UK studies found around 60% of early readmissions were due to the same reason as the primary admission, suggesting that these could have been reduced by medication reviews, better discharge communication and a rapid response to preventable issues.[6][7]

Both polypharmacy and chronic conditions such as chronic obstructive pulmonary disease (COPD), cardiovascular disease (CVD) and diabetes have been found to be associated with readmission rates and increased needs following discharge.[8][9] Accurately identifying patients as high risk enables resources to be channelled specifically to these patients through supportive interventions, rather than providing for all patients, many of whom may not be at risk of readmission. Several predictive models have been developed in the UK such as PARR-30[10] and in Canada the LACE[11] with relatively good predictive accuracy.

Evaluating the effectiveness of interventions designed to prevent early readmissions is problematic due to the lack of robust studies with good methodologies.[9] Intervention types which have been studied, often in combination include: extensive discharge planning, telephone calls, home visits, a 24-hour hot line and patient education.[9] The provision of follow up telephone calls is a common

For peer review only

For peer review only - http://bmjopen.bmj.com/site/about/guidelines.xhtml

intervention, with variation in the number and length of calls and profession of caller. The most successful results included both pre and post-discharge interventions.[12]

Schemes for supporting patients with their medications in the community were introduced into community pharmacies in 2005. Medicine Use Reviews (MURs) and New Medicines Service (NMS) can support patients with medication adherence as well as identifying interactions and other problems. The NMS is specifically targeted at patients with long term conditions such as COPD to support patients starting a new medication.[13]

The need for successful management of the pre and post-discharge period is highlighted in the National Institute for health and Care Excellence (NICE) Guidelines[14], developed in 2015 to help with the transition of adult patients with social care needs from hospital to the community. These guidelines emphasise the importance of the transition of care being co-ordinated using good communication. All healthcare professionals (HCPs) involved with the care of the patient in hospital and the community, should be included in the communication loop, with all patients/carers being provided with a medication list and a care plan with a single HCP responsible for co-ordinating the discharge for both social and medical needs.

This paper reports on the first stage of the OPTIMAL project[15], funded by Innovate UK. The OPTIMAL project encompasses the development of a predictive risk model, together with a supportive post-discharge patient intervention with the aim of reducing early hospital readmission. Although the success of both predictive risk models and interventions to prevent hospital readmission have been developed and studied separately before, this is the first time, to our knowledge, that a predictive model and a preventative intervention have been integrated to support patients.

The aim of this study was to undertake a needs assessment to investigate any common characteristics of patients admitted more than one time to CUH in a period of 12 months and understand their experiences of both the discharge process and the immediate post-discharge

For peer review only - http://bmjopen.bmj.com/site/about/guidelines.xhtml

period. The study also sought to determine factors contributing to frequent admission (>2 in 12 months). This will assist in the development of an appropriate post-discharge intervention for patients identified at high risk of readmission.

METHODS

A cross-sectional study was carried out at Croydon University Hospital (CUH). Patients were considered for inclusion in the study if they met the following criteria: ≥18 years, a home address on the CUH database, experienced ≥ 2 admissions to CUH in the past 12 months (August 2014- July 2015). Paediatric, oncology and maternity patients were excluded from the study. CUH research and development (R&D) department using patient records identified a total of 2732 patients who met the inclusion criteria. To provide a confidence level of 95% and a confidence interval of 5%, the sample size was calculated as 337 patients. As a low response rate may be expected from postal survey, all 2732 patients were invited to complete the postal questionnaire (Supplementary File). An explanatory letter was sent with the questionnaire together with a pre-paid return envelope. The questionnaire was only made available in English and no reminders were sent.

Ethical approval was obtained from Kingston University Delegated Research Ethics Committee (Ref: 1415/035) and approved by the R&D department by CUH as a service evaluation.

A quantitative cross-sectional questionnaire survey was designed using a mixture of open and closed questions. The validated tools AUDIT-C (a brief alcohol screening tool used to identify alcohol dependency)[15] and a medical health literacy score[16] were incorporated together with other questions which investigated patient experience and knowledge of medication and discharge counselling. The questionnaire was in four sections: Firstly, demographic information, collecting personal information such as age, as well as medication list and current medical conditions. Secondly, understanding the patient's admission experience, the reason for the patient's attendance at A&E and satisfaction with the admission process. Thirdly, the patient's discharge experience,

For peer review only

For peer review only - http://bmjopen.bmj.com/site/about/guidelines.xhtml

investigating patients' involvement in their discharge planning and the provision of medication counselling. Finally, understanding the patients' post-discharge experience, the discharge support received by patients, as well as patients' confidence in managing their health and coping at home post-discharge. The experience sought was based on the patient's most recent admission.

Pilot

After receiving ethics approval, a pilot study was conducted which involved asking 10 patients from the discharge lounge at CUH to complete the survey for validation. Minor changes were made to the questionnaire. To prevent any bias, the findings from the pilot were not included in the final results.

Patient and Public Involvement

The study was a follow up study from 50 patients at the Trust who indicated mixed experience in counselling and shared decision making during admission. As part of the funding, the researchers agreed to inform patients/public of the outcome of the study. This was completed via the public engagement forums within the Trust.

Data Analysis

The responses from the returned questionnaires were analysed using IBM SPSS ver. 23® through descriptive statistics and the Chi-squared test for independence, with a level of significance set at 5% ($p<0.05$). A comorbidity polypharmacy score (CPS) was calculated (defined as the total of the number of pre-trauma comorbidities and the number of pre-admission medications in trauma patients ≥45 years). Our modified calculation was performed for all patients ≥45 years, using the number of medications specified in the questionnaire, together with the number of existing comorbidities recorded. A three question Audit-C score[15] was calculated, with each question having a possible score of 0-4 and giving a total score in the range between 0-12. A score of ≥ 5 is considered positive, indicating a higher risk of alcohol consumption. A single question health literacy tool was utilised giving scores of 1-5, with scores >2 indicating some difficulty reading printed health

For peer review only - http://bmjopen.bmj.com/site/about/guidelines.xhtml

Parameter	Number (n,%)	Mean (SD)	Range	Mode
**Age (n=334)**	334, 100.0%	69.2 (18.2)	18-100	84
**Gender (n=337)**				
Male	155, 46.0%			
Female	182, 54.0%			
**Ethnicity (n=333)**				

material. The number of medications most associated with adverse drug reactions (ADR) resulting in hospital admission was also recorded for each patient.[17]

A linear regression analysis was carried out on the data to help identify significant patient characteristics which may have contributed to a greater number of admissions in the previous 12 months. This was carried out by adding a dependent variable column "frequent_admitter" to the data which was then assigned 1 if a patient's admissions in the previous year were >2 or 0 if ≤2. The independent variables included in the regression analysis were: admission reason, ethnicity, condition complexity indicator (which was set if a patient described their existing situation as complex/complicated or reported ≥ 2 conditions), a care indicator (identified by patients who were in receipt of some home care), CPS, patient age, number of medications. Any row where any of these variables were missing was excluded, thus leaving 169 patients to be included in the regression analysis.

RESULTS

The questionnaires were sent to 2722 patients, 347 were completed and returned giving a response rate of 12.7%.

The most common reasons given for the last admission were respiratory problems such as asthma and COPD (15.0%, n=52). Nearly 10% (n=33) of patients were admitted due to a fall. Nearly a third (n=101) of patients reported more than one condition or described their condition as complex (Table 1).

Table 1 *Demographics and Medical Conditions of responders*

For peer review only

For peer review only - http://bmjopen.bmj.com/site/about/guidelines.xhtml

White	250, 75.1%			
Black	34, 10.2%			
Chinese	3, 0.9%			
Mixed	9, 2.7%			
Asian	25, 7.5%			
Other	9, 2.7%			
Prefer not to say	3, 0.9%			
**Medical History**				
No. of admission in previous 12 months*[1]	347, 100.0%	2.8 (1.9)	2-30	2
No. of admission in previous 30 days*[1]	32, 10.8%	1.4 (0.9)	0-6	1
**Most Common Reason for Last Admission (n=347)**				
Respiratory Conditions	52, 15.0%			
Chest Pain	18, 5.2%			
Other Pain	20, 5.8%			
Fall	33, 9.5%			
Infections excl. Chest	28, 8.1%			
Cardiac Conditions	23, 6.2%			
Other	132, 38.0%			
Not Specified	41, 11.8%			
**Most Common Existing Medical Conditions (n=347)**				
Cardiac Conditions	59, 17.0%			
Respiratory Conditions	52, 15.0%			
Hypertension	41, 11.8%			
Diabetes	42, 12.1%			
None Specified	123, 35.4%			
>1 Long Term Condition (LTC) or Described as Complex	101, 29.1%			

*[1] Number of patients admitted within previous 12 months and 30 days. Mean (SD), Range and Mode reflect number of admissions per patient sample.

Over a quarter (28.8%, n=99/344) of patients lived alone and less than 5% (4.4% n=15/344) lived in a care home. Not all patients had someone to care for them; 26.7% (n=88/330) reported that they had no available care. Only 13.1% (n=43/328) of patients currently smoked, which is less than the

For peer review only

For peer review only - http://bmjopen.bmj.com/site/about/guidelines.xhtml

UK average of 19%.[18] However, 39.3 % (n=129/328) described themselves as ex-smokers. Nearly a third of patients had a limited health literacy score (29.8%, n=101/339) and over 15% (16.6%, 30/180) had a positive AUDIT-C score associated with a higher alcohol consumption risk.

Admission

Over half of patients were referred to A&E by an HCP (58.8%, n=204/347), with just over a third (34.6%, n=120/347) of patients reporting that a family member or they themselves made the decision. Although, two-thirds of patients (67.4%, n=234/347) were consulted regarding admission and care decisions, most patients (89.6%, n=311/347) wanted to be more involved with these decisions. The most frequently expressed comments about the admission experience concerned communication problems and the lack of provision of information (41.1%, n=35/85).

Regression Analysis

Four variables were found to be significantly associated with >2 admissions in the previous 12 months. These were admission for a fall (p=0.01), not identifying as having a complex condition or reporting <2 conditions (p=0.003), age (p=0.008) and being in receipt of care at home (p=0.005). Additionally, the overall regression is significant according to the F test (F=0.04). These factors were still significant for the sample when analysing only those patients ≥55years of age (F=0.007). The only change was that admission due to infection became significant in this sample (p=0.002). For patients with admissions >3 in 12 months CPS was found to be an additionally significant factor (p=0.002).

Discharge

Nearly half of patients, (42.1%, n=146/347) were not informed of the discharge decision 24 hours in advance, including 43.4% (n=43/99) of those who lived alone.

For peer review only - http://bmjopen.bmj.com/site/about/guidelines.xhtml

Over half of patients, (54.0%, n=187/347) were discharged from the hospital on a weekday between 12 noon and 6pm. However, about a quarter of patients (21.3%, n=74/347) were discharged between 6pm-6am with 17.6% (n=13/74) of them living alone with an average age of 71.2 years.

Two thirds (67.4% n=234/347) of patients agreed that the decisions regarding the discharge procedure were clearly explained (Table 2). However, only a third of patients (34.3%, n=119/347) were provided with information to enable them to detect signs of deteriorating health. Furthermore, only a third of patients (33.4% n=116/347) were provided with contacts for out-of-hours support. Less than a third of patients were referred to a post-discharge service and less than half of respondents reported joining this service (Table 2).

Table 2 *Patients' Discharge Experience*

Patient Discharge Experience (n=347)	Number (n,%)
Received discharge information from a doctor	188, 54.2%
Felt the decisions at discharge were clearly explained	234, 67.4%
Was fully consulted in the decision of being discharged	226, 65.1%
Received a written copy of care plan	146, 42.1%
Told about signs or signals to watch out indicating health was worsening	119, 34.3%
Told who to contact if health deteriorated	84, 24.2%
Told who to contact for out of hours help	116, 33.4%
Referred to a post-discharge service	95, 27.4%
Patient joined the post-discharge service (**n=95**)	46, 48.4%
Provided with details of local support groups	63, 18.2%

When patients were asked their opinion about their discharge procedure, 72 patients responded. The main concerns expressed were the poor provision of information and communication difficulties at all levels. Patients' concerns included the lack of communication between hospital staff and the patients/patients' families (48.6% n=35/72), including two elderly patients discharged without

For peer review only - http://bmjopen.bmj.com/site/about/guidelines.xhtml

informing their families. One patient stated: "more co-ordination is needed between the pharmacy and wards." Patients were also concerned about long waiting times (36.1% n=26/72), with 42.3% (n=11/26) of the waiting times involving a delay in receiving medications.

Medications

Two-thirds of patients reported taking at least one regular medication (67.4%, n=234/347). Three-quarters of these patients experienced changes to their medications whilst in hospital (75.2%, n=176/234), but over a quarter of these patients (28.4%, n=50/176) did not receive any counselling. Over two-thirds of patients (70.5%, n=165/234) agreed that medication information was explained in a way they could understand. However, 34.6% (n=81/234) would have liked more information regarding their medications.

The average number of medications per patient was 4.2 with of a range of 0 to 25. Nearly two-thirds (65.0%, n=152/234) of patients were taking $\geq$ 5 medications. The most commonly prescribed medication classes are shown in Table 3.

Table 3 *Most Common Medication Classes*

**Medication Class (n=234)**	**Number (n,%)**
Proton Pump Inhibitors	107, 45.3%
Statins	105, 44.5%
Antiplatelet drug	84, 35.6%

For peer review only - http://bmjopen.bmj.com/site/about/guidelines.xhtml

ACEI/ARBs (Angiotensin Converting Enzyme Inhibitors/ Angiotensin Receptor Blockers)	80, 33.9%
Beta Blockers	80, 33.9%
Calcium Channel Blockers	65, 27.5%
Loop Diuretics	52, 22.0%
Opioid analgesics (Including tramadol)	32, 13.6%
Oral Anti-Coagulants	34, 14.4%
B-2 Agonists	35, 14.8%

Some of the medication combinations found are not routinely recommended, due to being identified as risky.[19] For example, 10.7% (n=25/234) of patients were taking the high-risk combination of two or more anti-platelet drugs or an antiplatelet drug together with the anti-coagulant warfarin. Also 4.3% (n=10/234) were taking the high-risk triple combination of an (Angiotensin Converting Enzyme Inhibitors (ACEI)/ Angiotensin Receptor Blockers (ARB)), a Non-Steroidal Anti-Inflammatory Drug (NSAID) and a diuretic.

Over half of patients (56.4%, n=132/234) were prescribed two or more 2 medications that could put them at high risk of admission due to an ADR (Table 4).

Table 4 *High Risk Drugs*

**Number of High-Risk Meds (n=234)**	**Number (n,%)**
>5	2, 0.9%
5	15, 6.4%
4	14, 5.9%
3	49, 20.8%
2	52, 22.0%
1	52, 22.0%
0	50, 22.0%

The average calculated CPS score was 7.5. Scores greater than 7 are associated with an increased risk of falls and length of hospital stay, complications, short term and one year mortality [20], over 40% of patients (n=135/313) had scores >7 and 20 patients were considered as severe or morbid with scores between 15 and 32.

For peer review only - http://bmjopen.bmj.com/site/about/guidelines.xhtml

Post-Discharge Experience

While 70.3% (n=244/347) of patients were confident in managing their own health, 41.8% (n=145/347) had concerns about being readmitted to the hospital, with two patients feeling that their last admission was due to medicine errors that could have been avoided. Receiving medication counselling in hospital (55.1%, n=191/347) was significantly associated with patients feeling more confident in the management of their health care issues (p=0.013). Three-quarters of patients (74.9% n=260/347), were confident in managing their supply of medicines, but were less confident in managing their social care issues (34.3%, n=119/347) and healthcare issues (48.9%, n=170/347).

Almost half of patients (46.9% n=163/347) were very satisfied or satisfied with the available support post-discharge in managing their health needs. However, less than a third of patients were satisfied (27.4% n=95/347) with the support for their social care needs.

During the crucial first 30 days post-discharge from hospital, over half (54.5%, n=189/347) of patients did not receive any contact from a hospital, GP, pharmacy, or other post-discharge service. Only 17.6%, (n=61/347) of patients reported being contacted by their GP. During this time, patients were also very reticent to contact an HCP themselves, with only 12.1% (n=42/347) of patients reporting initiating contact.

Just under a quarter of patients (24.2% n=84/347) were contacted by other post-discharge services, of the 58 patients who specified a service, half were contacted by community or other nurse, but only 15% (n=3/20) of patients suffering from COPD, 13 of which were admitted with a respiratory problem/exacerbation, were referred to the respiratory HOT clinic (a rapid access clinic to help patients with COPD avoid hospitalisation).[21]

Community pharmacy support services were not well utilised post-discharge and only 4.0%, (n=17/347) of patients were referred to MUR, with 69.7% (n=242/347) of patients being unaware of MUR services. However, 50.4% (n=175/347) of patients were interested in receiving this service.

For peer review only - http://bmjopen.bmj.com/site/about/guidelines.xhtml

Similarly, 78.9% (n=274/347) of patients were not referred to NMS, with 51.6% (n=179/347) of patients being interested in receiving this service.

DISCUSSION

This questionnaire-based study followed patients that had ≥2 hospital admissions/year living in the vicinity of CUH from admission, through discharge to post-discharge. Despite the low response rate, this is the first study that captures the complete patient journey from admission, discharge, through to post-discharge care. Furthermore, it identified characteristics of patients with high admission rates. A strength of this is the holistic nature of the reported data, which provides a comprehensive picture of these patients' experience of the support they were given, their physical health and medication when discharged from hospital. The data highlights a wide range of areas for improving patient support, including communication, utilisation and integration of services and medication counselling.

The study had several limitations: Firstly, it is representative of the population around CUH and admissions to that Trust only, as well as being limited by the memory of the respondents. Secondly, not all patients fully completed the questionnaire, hence, statistical significance was not achieved for the whole questionnaire. Thirdly, as the questionnaire was only available in English, this limited the study to participants who had sufficient English, the black population was also under represented at 10.2% compared to the 2011 census figure of 20.2%.[22] Three quarters (75.1%) of patients described themselves ethnically as white, which is an over representation when compared to the Croydon borough 2011 census figure of 47.3%.[22]

Regression analysis identified four patient characteristics associated with higher admission. It is interesting that two of these factors: falls and being in receipt of care, both require liaison with other services post-discharge to provide adequate support in the patient's home. Suffering from falls is a well-known cause of hospital admission and corroborates with other studies[23][24], but being in receipt of care is, as far as we are aware a novel, though not surprising reason for admission. Falling

For peer review only - http://bmjopen.bmj.com/site/about/guidelines.xhtml

was the second most common reason for admission as reported by nearly 10% of patients. Polypharmacy, higher CPS score and identifying one's condition as complex or having >2 existing conditions were not significantly associated with >2 admissions in 12 months. However, a higher CPS score was found to be a significant contributor to high levels of admission (>3 in 12 months). Medications may often be implicated in falls with an increased risk for patients even those taking < 5 medications, however the medication class may be deemed to be more significant than the number.[25][26] Nevertheless, a higher CPS has been associated with an increase in falls by other studies, which may explain why this study found this factor to be significant for those that had >3 admissions in 12 months.[27][28] Nearly 50% of patients had a CPS score ≥ 7 and 65% were taking 5 or more medications. An Australian study observed a median increase from 3 to 6 annual attendances in the emergency department (ED) for those ≥65 years old who presented with comorbidities and polypharmacy (≥5 medications), among other factors.[28]

There is additional evidence to suggest that co-morbidities are a significant factor when predicting early readmission. The Charlson Index, which predicts 10-year mortality based on patients' comorbidities, was found to be significantly associated with readmission within 28 days for patients scoring ≥3 in a retrospective observational study by Li et al.[29] Interestingly, Considine et al[30] found that comorbidities were not significant predictors of readmission ≤1 day post-discharge for patients from acute-care, however health service use was notable in the 6-months preceding the index admission with ≥1 ED attendance or ≥1 hospital admission in 42.6% (n=579) and 40.7% (n=553) respectively. Although our study focused primarily on frequent admission as opposed to readmission, the latter study could provide an explanation of why co-morbidities were only a predictor of high admission rate (>3 in 12 months).[30]

It must be noted that in this study, medications and conditions were self-reported. However, these were not found to be significantly associated with frequent admission (>2 in 12 months), thus

For peer review only - http://bmjopen.bmj.com/site/about/guidelines.xhtml

highlighting that social care needs are superseding medical needs in determining increased admission risk with medical needs becoming significant in those with >3 admissions in 12 months.

Receipt of medication counselling was significantly associated with patient confidence in managing health (p=0.013). Medicine combinations were reported which could have been questioned, such as patients taking two anti-platelet drugs or an anti-platelet drug with warfarin, which can lead to an increase risk of bleeding.[31] Ten patients were taking the combination of NSAID, ACEI/ARB together with a diuretic, this combination is associated with an increased risk of acute kidney injury.[32] Community pharmacists being the most accessible HCP, are well placed to identify medications which cause adverse events to patients and increase their risk of falls. Patients were not referred to and had a lack of awareness of community pharmacy medicine information schemes - MUR or NMS. This was a missed opportunity for medication support post-discharge in the community. In fact, an initiative at CUH that piloted the provision of domiciliary MUR to housebound 'high risk' patients by community pharmacists resulted in reported avoidance of hospitalisation.[33][34]

Although nearly three quarters of patients felt consulted in the decisions leading to their discharge, patients expressed dissatisfaction with the discharge process, with long waiting times, delays and poor communication reported as the most common complaints. These findings correlate with an AGE UK report[35] investigating older people's experience of hospital readmission. Delays in discharge and lack of information are upsetting and confusing. Patients should at least be provided with updates as to the progress of their discharge. Although this study is limited to the experiences of the population around Croydon, a study from Liverpool Hospital UK[36] reported similar percentages of patients (70%) who felt that discharge decisions were explained, with the long wait for discharge medications also having a negative influence on the discharge experience.

Nearly 50% of patients were worried about being readmitted to hospital and commented on finding the experience stressful and wanting to avoid readmission. Good communication and information

For peer review only - http://bmjopen.bmj.com/site/about/guidelines.xhtml

sharing supports the transition from hospital and helps prevent readmission.[14][37] Contact information should be provided in case of a short-term crisis, which should be proactive rather than waiting for a more serious problem to arise. However, it was found that nearly 40% of patients were not provided with the signs of deterioration of their condition and nearly three quarters of patients were not provided with details of who to contact if this situation arose. This lack of information could result in patients returning to hospital. Additionally, patients' carers and families were not always informed of the discharge, making it hard for them to adequately support the patient at home.

Poor integration of services was found both within the hospital and between primary and secondary care providers. Patients with social care needs should be contacted by a GP or community nurse within 24-72 hours of discharge.[14] However, less than 20% of patients were contacted by their GP within 30 days of discharge. A further 12.1% contacted a HCP themselves. Additionally, patients were not being referred to post-discharge services which could have supported them. Despite 20 patients reporting suffering from COPD and 13 of these patients reporting respiratory problems/exacerbation as the reason for admission, only 3 patients were referred to the respiratory HOT clinic at CUH[21] which provides an integrated team of multidisciplinary HCPs. Nearly a third of patients were dissatisfied with their social care, thus it is not surprising that those receiving care were more at risk of frequent admission. A lack of transition of care was reported, with a need for low level practical support during the first few days after discharge. This is a shared outcome with the AGE UK report.[35]

More integrated support such as that provided by Lewisham Integrated Medicines Optimisation Scheme (LIMOS)[38] can break through traditional boundaries of care, but as these authors note such links with services take time to build. With an increasing aging population with more multi-morbidities, the integration of service delivery across different clinical areas becomes more important to provide appropriate individual care, rather than the current disease-focused

For peer review only - http://bmjopen.bmj.com/site/about/guidelines.xhtml

practice.[39] A move to a shared responsibility, is required across multiple areas- social, voluntary and clinical, to provide the integrated personalised care that patients need.[40]

CONCLUSIONS

The study highlighted gaps in care during the patient discharge journey. Admission for a fall and receipt of care were significantly associated with higher admission rates. Additionally, it reports for the first time, that social care is an important determinant of frequent admission (>2 in 12 months) in a predominantly older population. Before discharge, patients lacked medication counselling, information on symptoms of deteriorating health, or HCP to contact if this situation arose. An improvement in communications at all levels would benefit patients, ensuring patients are informed of delays and decisions. Additionally, patients' confidence in their care being well managed may be increased by demonstrating that communication channels are open between different HCPs. Post-discharge, patients were lacking referrals to relevant services which could have supported them. The study highlighted that transitional care is fragmented between different services of primary, secondary and social care as well as the voluntary sector. This lack of integration is causing patients avoidable difficulties. Improvement could be made by increasing HCP awareness of the available services, both voluntary and statutory, in the local area and encouraging links. Integrating services would increase the utilisation of existing resources, such as community pharmacy medicine support schemes, hospital services, e.g. respiratory HOT clinics as well as voluntary services, with care pathways utilising all relevant services across each sector.

CONTRIBUTORSHIP STATEMENT RK was the principal investigator of the study. She was responsible for the design of the study. She also organised and co-ordinated all aspects of this research. GF worked alongside RK to draft the publication. BO contributed to data collection. The analysis of the results was carried out by RK, GF, BP, SNG, YK, AS, JW and JC.

For peer review only - http://bmjopen.bmj.com/site/about/guidelines.xhtml

COMPETING INTERESTS All authors have completed the ICMJE form for disclosure of potential conflicts of interest available from www.icmje.org/coi_disclosure.pdf and declare that there is nothing to disclose.

FUNDING: This research was carried out as part of the OPTIMAL project which has received funding from Innovate UK.

DATA SHARING STATEMENT No additional data is available

1. OECD Health Ministerial Meeting: Health System Priorities in the Aftermath of the Crisis [Internet]. 2010 [cited 2017 Feb 7]. p. 7–8. Available from: https://www.oecd.org/health/2010-ministerial/46098360.pdf

2. National Audit Office. Emergency admissions to hospital: managing the demand [Internet]. Vol. 739, Hc. 2013 [cited 2017 Feb 10]. p. 4. Available from: https://www.nao.org.uk/wp-content/uploads/2013/10/10288-001-Emergency-admissions.pdf

3. Department of Health. Payment by Results Guidance for 2011-12 [Internet]. 2011 [cited 2017 Feb 17]. Available from: http://webarchive.nationalarchives.gov.uk/20130507170152/https://www.gov.uk/government/uploads/system/uploads/attachment_data/file/151911/dh_126157.pdf.pdf

4. Benbassat J, Taragin M. Hospital Readmissions as a Measure of Quality of Health Care. arch intern med. 2000;160:1074–81.

5. Lindquist LA, Baker DW. Understanding preventable hospital readmissions: Masqueraders, markers, and true causal factors. J Hosp Med. 2011;6(2):51–3.

6. Conroy SP et al. Understanding readmissions: An in-depth review of 50 patients readmitted back to an acute hospital within 30 days. Eur Geriatr Med [Internet]. 2013;4(1):25–7. Available from: http://dx.doi.org/10.1016/j.eurger.2012.02.007

7. Jakupaj A. Reducing readmission rate in the elderly population at a District General Hospital. Eur Geriatr Med [Internet]. 2014;5:S248–9. Available from: http://ovidsp.ovid.com/ovidweb.cgi?T=JS&PAGE=reference&D=emed12&NEWS=N&AN=71659663

8. Hurding S et al. Polypharmacy Guidance 2015 (NHS Scotland). 2015.

9. Jayakody A et al. Effectiveness of interventions utilising telephone follow up in reducing hospital readmission within 30 days for individuals with chronic disease: a systematic review. BMC Health Serv Res [Internet]. 2016;16(1):403. Available from: http://www.ncbi.nlm.nih.gov/pubmed/27538884%5Cnhttp://www.pubmedcentral.nih.gov/articlerender.fcgi?artid=PMC4990979

10. Billings J et al. Development of a predictive model to identify inpatients at risk of re-admission within 30 days of discharge (PARR-30). BMJ Open [Internet]. 2012;2(4):e001667–e001667. Available from: http://www.ncbi.nlm.nih.gov/pubmed/22885591%5Cnhttp://www.pubmedcentral.nih.gov/articlerender.fcgi?artid=PMC3425907

For peer review only - http://bmjopen.bmj.com/site/about/guidelines.xhtml

11. Spiva L et al. Validation of a Predictive Model to Identify Patients at High Risk for Hospital Readmission. J Heal Qual. 2015;00(0):1–7.

12. Sales VL et al. Utilization of Trained Volunteers Decreases 30-Day Readmissions for Heart Failure. J Card Fail [Internet]. 2014;20(5):377–377.e23. Available from: http://linkinghub.elsevier.com/retrieve/pii/S1071916414001432

13. NHS. Medicine Use Reviews [Internet]. [cited 2017 Apr 10]. Available from: http://www.nhsemployers.org/your-workforce/primary-care-contacts/community-pharmacy/medicines-use-reviews-murs

14. NICE NG27. NICE Transition between inpatient hospital settings and community or care home settings for adults with social care needs NG27 [Internet]. 2015 [cited 2017 Mar 5]. p. 1–29. Available from: https://www.nice.org.uk/guidance/ng27

15. EXUS Innovations. OPTIMAL project [Internet]. [cited 2017 Apr 10]. Available from: https://www.exusinnovation.co.uk/en/optimal/

16. Bush K et al. The AUDIT Alcohol Consumption Questions (AUDIT-C) An Effective Brief Screening Test for Problem Drinking. Arch Intern Med. 1998;158(16):1789–95.

17. Chew LD et al. Validation of screening questions for limited health literacy in a large VA outpatient population. J Gen Intern Med. 2008;23(5):561–6.

18. Pirmohamed M et al. Adverse drug reactions as cause of admission to hospital: prospective analysis of 18 820 patients. Bmj. 2004;329(7456):15–9.

19. Centre H and SCI. Statistics on Smoking - Key facts [Internet]. 2016 [cited 1917 Jan 9]. Available from: http://content.digital.nhs.uk/catalogue/PUB20781/stat-smok-eng-2016-app.pdf

20. All Wales Medicines Strategy Group. Polypharmacy: Guidance for Prescribing. July 2014. [Internet]. 2014 [cited 2017 Mar 7]. Available from: http://www.awmsg.org/docs/awmsg/medman/Polypharmacy - Guidance for Prescribing.pdf

21. Housley BC et al. Comorbidity-polypharmacy score predicts readmission in older trauma patients. J Surg Res [Internet]. 2015;199(1):237–43. Available from: http://dx.doi.org/10.1016/j.jss.2015.05.014

22. Croydon Health Care Trust. HOT Clinics [Internet]. 2012 [cited 2017 Apr 19]. Available from: https://www.croydonhealthservices.nhs.uk/new_page.htm

23. UK Office For National Statistics. 2011 UK Census Aggregate Data [Internet]. 2011 [cited 2017 Apr 10]. Available from: https://www.ons.gov.uk/census/2011census

24. Hughes LD, Witham MD. Causes and correlates of 30 day and 180 day readmission following discharge from a Medicine for the Elderly Rehabilitation unit. BMC Geriatr. 2018;18(1):1–10.

25. Hallgren J, Aslan AKD. Risk factors for hospital readmission among Swedish older adults. Eur Geriatr Med [Internet]. 2018;9(5):603–11. Available from: https://doi.org/10.1007/s41999-018-0101-z

26. Helgadóttir B et al. Medication and fall injury in the elderly population; Do individual demographics, health status and lifestyle matter? BMC Geriatr. 2014;14(1):1–8.

27. Fritsch MA, Shelton PS. Geriatric Polypharmacy: Pharmacist as Key Facilitator in Assessing for Falls Risk. Clin Geriatr Med. 2017;33(2):205–23.

For peer review only - http://bmjopen.bmj.com/site/about/guidelines.xhtml

28. Berry D, Street M, Considine J. Service use by older very frequent emergency department users: A retrospective cohort study. Australasian emergency care. 2019 Sep 1;22(3):133-8.

29. Li JY, Yong TY, Hakendorf P, Ben-Tovim DI, Thompson CH. Identifying risk factors and patterns for unplanned readmission to a general medical service. Australian Health Review. 2015 Feb 24;39(1):56-62.

30. Considine J, Fox K, Plunkett D, Mecner M, O'Reilly M, Darzins P. Factors associated with unplanned readmissions in a major Australian health service. Australian Health Review. 2019 Feb 18;43(1):1-9.

31. Lee YM et al. Impact of health literacy on medication adherence in older people with chronic diseases. Collegian [Internet]. 2017;24(1):11–8. Available from: http://dx.doi.org/10.1016/j.colegn.2015.08.003

32. Lapi F et al. Concurrent use of diuretics, angiotensin converting enzyme inhibitors, and angiotensin receptor blockers with non-steroidal anti-inflammatory drugs and risk of acute kidney injury: nested case-control study. BMJ [Internet]. 2013;346(jan08_12):e8525. Available from: http://www.bmj.com/content/346/bmj.e8525

33. Jesson B, Williams V. Domiciliary Medicine Review Service in Croydon - Presentation [Internet]. Pharmacy Management National Forum - Forum Workshop. 2015 [cited 2017 Apr 7]. Available from: http://www.pharman.co.uk/imagelib/pdfs/Domiciliary_Medicine_Review_Service_in_Croydon_-_Barbara_Jesson_and_Victoria_Williams.pdf

34. Williams, V Jesson, B McCoig, A Beavon, N Rajah D. Domiciliary MURs: our experience in Croydon [Internet]. Pharmaceutical Journal. 2012 [cited 2017 Apr 9]. Available from: http://www.pharmaceutical-journal.com/opinion/correspondence/domiciliary-murs-our-experience-in-croydon/11109461.article

35. Lawrie M, Battye F. Older people's experience of emergency hospital readmission [Internet]. Age UK. 2012 [cited 2017 Mar 9]. Available from: http://www.ageuk.org.uk/Documents/EN-GB/For-professionals/Research/Emergency_readmission_older_peoples_experiences.pdf?dtrk=true

36. Wright S et al. UK hospital patient discharge: the patient perspective. Eur J Hosp Pharm. 2017;(January).

37. Winfield A, Burns E. Let's all get home safely : a commentary on NICE and SCIE guidelines (NG27) transition between inpatient hospital settings and community or care home settings. Age Ageing. 2016;45(August):757–60.

38. Lai K et al. Lewisham integrated medicines optimisation service: delivering a system-wide coordinated care model to support patients in the management of medicines to retain independence in their own home. Eur J Hosp Pharm. 2015;22(2):98–101.

39. Stange KC, Ferrer RL. The Paradox of Primary Care (editorial). Ann Fam Med [Internet]. 2009;7(4):293–9. Available from: https://www.ncbi.nlm.nih.gov/pmc/articles/PMC2713149/pdf/0060293.pdf

40. Valentijn PP et al. Understanding integrated care: a comprehensive conceptual framework based on the integrative functions of primary care. Int J Integr Care. 2013;13(March).

For peer review only

For peer review only - http://bmjopen.bmj.com/site/about/guidelines.xhtml

Section A: YOUR ADMISSION EXPERIENCE

*This part is about your experience while you were being **admitted and treated** at Croydon University Hospital.*

1) What was the reason for your last admission at Croydon University Hospital?

2) Who decided that you need to go to A&E?

- ☐ Self
- ☐ Family
- ☐ GP
- ☐ Ambulance paramedic
- ☐ I don't know
- ☐ Other *(please specify)*

3) Did you try to seek help from any of the following before attending A&E? *(please select all that apply)*

- ☐ None. I went directly to A&E
- ☐ Calling 111
- ☐ Contacting GP
- ☐ Visiting Walk-in centre
- ☐ Self-Care from the Pharmacy
- ☐ Community nurses
- ☐ HOT Clinics
- ☐ Other *(please specify)*

4) What day/time did you arrive to A&E?*(please select one box only)*

Day/Time	6am-12noon	12noon - 6pm	6pm-12midnight	12midnight - 6am
Weekday (Mon-Fri)	☐	☐	☐	☐
Weekend (Sat-Sun)	☐	☐	☐	☐

5) Following your arrival at A&E, how long did you wait before you were examined by a doctor?

- ☐ 1-2 Hours
- ☐ 2-3 Hours
- ☐ 3-4 Hours
- ☐ > 4 Hours *(please specify)*

6) Following examination by a doctor, how long did you wait before you were admitted to a bed in the ward?

- ☐ 1-2 Hours
- ☐ 2-3 Hours
- ☐ 3-4 Hours
- ☐ > 4 Hours *(please specify)*

7) To what extent do you agree/disagree that the following were explained to you in a way you could clearly understand

i The reasons for your admission

- ☐ Strongly Disagree
- ☐ Disagree
- ☐ Neutral
- ☐ Agree
- ☐ Strongly Agree

ii The decisions regarding your care, treatment, and/or procedure

- ☐ Strongly Disagree
- ☐ Disagree
- ☐ Neutral
- ☐ Agree
- ☐ Strongly Agree

8) Were you consulted regarding the decisions about your care, treatment, and/or procedure on admission?

- ☐ Yes
- ☐ No
- ☐ Can't Remember

9) In the future, would you like to be involved in decisions about your care, treatment, or procedure?

- ☐ Yes
- ☐ No
- ☐ Can't Remember

10) What could have improved your experience while being admitted to the hospital?

PROPRIETARY RIGHTS STATEMENT
This document contains information, which is proprietary to the OPTIMAL Consortium. Neither this document nor the information contained herein shall be used, duplicated or communicated by any means to any third party, in whole or in parts, except with prior written consent of the OPTIMAL consortium.

For peer review only

Section B: YOUR DISCHARGE EXPERIENCE

The following part is about your experience while you were being ***discharged from the hospital****.*

1) Were you informed 24 hours in advance about the discharge decision?
- ☐ Yes
- ☐ No
- ☐ Can't Remember

2) What day/time were you discharged from the hospital? *(Please tick one box only)*

Day/Time	6am-12noon	12noon-6pm	6pm-12midnight	12midnight-6am
Weekday (Mon-Fri)	☐	☐	☐	☐
Weekend (Sat-Sun)	☐	☐	☐	☐

3) Who provided you with the information related to your discharge? *(please select all that apply)*
- ☐ Doctor
- ☐ Nurse
- ☐ Pharmacist
- ☐ No one
- ☐ Other (please specify)

4) To what extent do you agree/disagree with the following:

i The decisions regarding my discharge were explained to me in a way I could clearly understand
- ☐ Strongly Disagree
- ☐ Disagree
- ☐ Neutral
- ☐ Agree
- ☐ Strongly Agree

ii I was fully consulted with the decision of being discharged from the hospital
- ☐ Strongly Disagree
- ☐ Disagree
- ☐ Neutral
- ☐ Agree
- ☐ Strongly Agree

iii Staff took my preferences into account in deciding how my health care will be managed when I will leave the hospital
- ☐ Strongly Disagree
- ☐ Disagree
- ☐ Neutral
- ☐ Agree
- ☐ Strongly Agree

5) Was there any change in your medicines during your last hospital admission?
- ☐ Yes
- ☐ No
- ☐ Can't Remember

6) Were you provided with any counselling about your medication(s)?
- ☐ Yes (go to 7)
- ☐ No (go to 12)
- ☐ Can't Remember

7) Who provided you with the information related to your medication(s) at discharge?
(please select all that apply)
- ☐ Doctor
- ☐ Nurse
- ☐ Pharmacist
- ☐ Other please specify

8) How was this information given to you?
- ☐ Verbally
- ☐ Written
- ☐ Verbally & Written

9) What resources were you given to help you take your medicine(s)? *(please select all that apply)*
- ☐ Patient information leaflet in box
- ☐ Medication reminder card
- ☐ Medication record book
- ☐ Poster or brochure
- ☐ None
- ☐ Other (please specify)

10) To what extent do you agree/disagree with the following:

i The information about my medication(s) were given/explained to me in a way I could clearly understand
- ☐ Strongly Disagree
- ☐ Disagree
- ☐ Neutral
- ☐ Agree
- ☐ Strongly Agree

ii I would like to have more information regarding my medicines
- ☐ Strongly Disagree
- ☐ Disagree
- ☐ Neutral
- ☐ Agree
- ☐ Strongly Agree

For peer review only - http://bmjopen.bmj.com/site/about/guidelines.xhtml

11) Please rate your satisfaction with regards to the following information about your medication, if provided during the counselling session:

Please tick one box for each row	Very Unsatisfied	Unsatisfied	Neutral	Satisfied	Very Satisfied	Not provided
Purpose of your medicine(s)	☐	☐	☐	☐	☐	☐
How to take/use the medicine(s)	☐	☐	☐	☐	☐	☐
Important side effects	☐	☐	☐	☐	☐	☐
Actions to take if you get any important side effects.	☐	☐	☐	☐	☐	☐
Lifestyle changes associated with taking your medicine(s)	☐	☐	☐	☐	☐	☐

For peer review only

For peer review only - http://bmjopen.bmj.com/site/about/guidelines.xhtml

12) Community pharmacies are offering a New Medicines Service (NMS) which is an open conversation between you and the pharmacist to discuss any concerns you may have about your new medicine(s) - for example side effects

i Are you aware of this service?
- Yes
- No

ii Were you offered/referred to this service upon discharge?
- Yes
- No
- Not Sure

iii Would you have been interested to be referred to this service?
- Yes
- No
- Not Sure

13) Community pharmacies are offering a Medicines Use Review (MUR) which is an open conversation between you and the pharmacist to discuss your medications after you were discharged from the hospital or periodically.

i Are you aware of this service?
- Yes
- No

ii Were you offered/referred to this service upon discharge?
- Yes
- No
- Not Sure

iii Would you have been interested to be referred to this service?
- Yes
- No
- Not Sure

14) When discharged from the hospital, were you given a written copy of your care plan?

☐ Yes
☐ No *(go to 15)*

i If yes, did you understand what was in this care plan?

☐ Yes
☐ No

15)* Were you referred to a post-discharge service? (e.g. hospital avoidance team, hot clinics, telehealth, community services nurse, social care)*

☐ Yes
☐ No *(go to 16)*

i If yes, please specify

ii Were you offered a choice to select those services?

☐ Yes
☐ No

iii Have you joined any of these services yet?

☐ Yes
☐ No

16) Were you told about signs/signals of worsening or decline of your health to watch out for?

☐ Yes
☐ No *(go to 17)*

i If yes, were you given details of who to contact if this happened?

☐ Yes
☐ No

17) Were you given contacts for out-of-hours help?

☐ Yes
☐ No

For peer review only - http://bmjopen.bmj.com/site/about/guidelines.xhtml

18) **Were you given information about local support groups?** *(E.g. Diabetes UK, Age UK, Cardiac Support Group, Breathe Easy groups, etc…)*

- ☐ Yes
- ☐ No

19) What could have improved your experience while being discharged from the hospital?

For peer review only

Section C: YOUR POST-DISCHARGE EXPERIENCE

*This part is about your experience since your **last hospital discharge***

1 How confident are you in managing your health?

- ☐ Not at all confident
- ☐ Not confident
- ☐ Neither
- ☐ Confident
- ☐ Completely Confident

i Why is that?

2 How worried have you been about being readmitted to the hospital?

- ☐ Very Worried
- ☐ Worried
- ☐ Neither
- ☐ Not Worried
- ☐ Not worried at all

i Why is that?

3 **Please rate your satisfaction with regards to the following**

Please tick one box for each row	Very Unsatisfied	Unsatisfied	Neutral	Satisfied	Very Satisfied	Not Applicable
The available support to manage your health after you were discharged from the hospital	☐	☐	☐	☐	☐	☐
Social care support after being discharged from the hospital *(e.g. help in your home, community support, etc…)*	☐	☐	☐	☐	☐	☐
Occupational needs support *(e.g. walking aids)*	☐	☐	☐	☐	☐	☐

4 When discharged from the hospital, were you given any follow up appointments?

- Yes
- No *(please go to 5)*
- Not Sure *(please go to 5)*

i If yes, who was the follow up appointment with?
(please select all that apply)

- GP
- Nurse
- Hospital Outpatient
- Pharmacist
- Other (please specify)

ii Were you able to attend all these appointments?

- Yes
- No

iii If no, why is that? (Optional)

iv To what extent do you agree/disagree with the following:

(a) I clearly understand my post-discharge care plan/appointments

- Strongly Disagree
- Disagree
- Neutral
- Agree
- Strongly Agree

(b) I could easily keep track/record of my appointments

- Strongly Disagree
- Disagree
- Neutral
- Agree
- Strongly Agree

(c) I prefer to have my appointments available for me electronically (e.g. smart phone calendar)

- Strongly Disagree
- Disagree
- Neutral
- Agree
- Strongly Agree

5 Have you received any follow up from the hospital?

- Yes
- No *(please go to 6)*

For peer review only - http://bmjopen.bmj.com/site/about/guidelines.xhtml

- Don't remember *(please go to 6)*

i If yes, how? *(please select all that apply)*
- By phone call
- By letter
- By email
- By text message (SMS)
- Other (please specify)

ii When?
- 1 week after discharge
- 2 week after discharge
- 3 week after discharge
- 4 week after discharge
- Other (please specify)

6 Have you been contacted by any other post-discharge service team? (e.g. hospital avoidance team, hot clinics, telehealth, community services nurse, social care)
- Yes
- No *(please go to 7)*
- Don't remember *(please go to 7)*

i If yes, who was it? (please specify)

ii How? *(please select all that apply)*
- By phone call
- By letter
- By email
- By text message (SMS)
- Other *(please specify)*

iii When?
- 1 week after discharge
- 2 week after discharge
- 3 week after discharge
- 4 week after discharge
- Other *(please specify)*

7 During the first 30 days after you were discharged from the hospital:

i Were you contacted by any of the following *(please select all that apply)*
- GP
- Hospital
- Pharmacy Team
- Post-discharge Service
- None
- Other *(please specify)*

ii Did you contact any of the following *(please select all that apply)*
- GP
- Hospital
- Pharmacy Team
- Post-discharge Service
- None
- Other *(please specify)*

8 How confident are you regarding the management of the following:

Please tick one box for each row	Not at all confident	Not confident	Neither	Confident	Completely Confident	Not applicable
Your supply of medicines	☐	☐	☐	☐	☐	☐
Your social care issues	☐	☐	☐	☐	☐	☐
Your healthcare issues	☐	☐	☐	☐	☐	☐

For peer review only

Section D: SOME INFORMATION ABOUT YOU

1) How many times were you admitted to the hospital

in the last 12 months?	
in the last 30 days?	

1) **In what year were you born?**

2) What is the first part of your postcode? (E.g. if your post code is CR7 7YE, please write CR7)

3) What is your gender?

□ **Male** □**Female**

4) How would you describe your ethnicity?

- White
- Black
- Chinese
- Mixed
- Asian
- Other
- **Prefer** not to say

5) What is/are the main language(s) spoken at your home?

6) How often do you need to have someone help you when you read instructions, pamphlets, or other written material from your doctor or pharmacy

- Never
- Rarely
- Sometimes
- Often
- Always

7) Do you suffer from any medical conditions? If yes please specify

8) How often do you have a drink containing alcohol?

- Never *(please skip to 11)*
- Monthly or less
- 2 to 4 times a MONTH
- 2 to 3 times a WEEK
- 4 or more times a week

9) How many drinks of alcohol do you drink on a typical day when you are drinking?

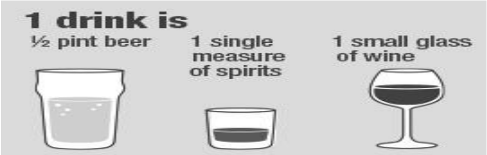

- 1 or 2 drinks
- 3 or 4 drinks
- 5 or 6 drinks
- 7 or 8 or 9 drinks
- 10 **or** more drinks

For peer review only

10) How often have you had 6 or more units on a single occasion in the last year?

- Never
- Less than monthly
- Monthly
- Weekly
- **Daily** or almost daily

11) Have you ever smoked a cigarette, a cigar or a pipe?

- Yes
- No *(please go to 12)*

i Yes, and I am currently a smoker

- How frequently do you smoke cigarettes?
  - Regularly
  - Occasionally
- (b) How many cigarettes do you smoke per day?
  - 10 or Less
  - 11-20
  - 21-30
  - 31 or More

ii Yes, but I am an ex-smoker

- How frequently did you smoke cigarettes?
  - Regularly
  - Occasionally
- (b) About how many cigarettes did you smoke in a day?
  - ☐ 10 or Less
  - ☐ 11-20
  - ☐ 21-30
  - ☐ 31 or More
- (c) For approximately how many years did you smoke cigarettes regularly?
- (d) How long ago did you stop smoking cigarettes?

iii Are you currently using e-cigarettes?

- ☐ Yes
- ☐ No

12) How many regular medicines are you currently taking?

13) How often do you miss doses of your medicine(s), cut down or stop taking them?

- ☐ Frequently (more than once a week)
- ☐ Occasionally (once a week)
- ☐ Rarely (once a month)
- ☐ Very rarely (once every 6 month)
- ☐ Never

14) Do you experience any of the following problems with your medicines which make it difficult for you to take your medicine(s)? (please select all that apply)

- ☐ Forget
- ☐ Cost
- ☐ Difficulty understanding the instructions
- ☐ Difficulty reading labels
- ☐ Difficulty opening containers

For peer review only

☐ Difficulty in administration *(e.g. difficulty in using Inhalers, swallowing tablets, or injection site, etc…)*

☐ Other *(please specify)*

15) Usually, how are your medicines supplied to you?

☐ Original or labelled box/bottle	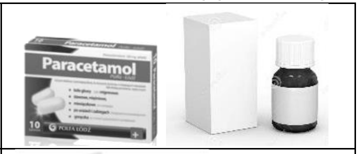
☐ Blister pack	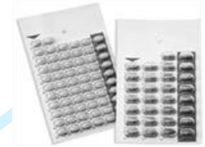
☐ Medicine Dosettes	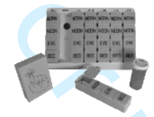

16) If known, please list your current medicines names

17) Who lives with you at home?

☐ I live alone

☐ I live with a family member

☐ I live with a friend

☐ I live in a care home

☐ Other please specify

18) Do you have someone who can take care of you?

☐ Yes

☐ No

i. Who provides you with home care?

☐ Family member

☐ Friend

☐ Carer

☐ Other please specify

ii. How many days a week are he/she/they available to you?

☐ < 1day

☐ 1-2 days

☐ 3-4 days

☐ 5-7 days

iii. When are they available to you?

For peer review only

- ☐ Weekdays only
- ☐ Weekend only
- ☐ Both

For peer review only

For peer review only - http://bmjopen.bmj.com/site/about/guidelines.xhtml

STROBE 2007 (v4) Statement—Checklist of items that should be included in reports of *cross-sectional studies*

Section/Topic	Item #	Recommendation	Reported on page #
**Title and abstract**	1	(*a*) Indicate the study's design with a commonly used term in the title or the abstract	1
		(*b*) Provide in the abstract an informative and balanced summary of what was done and what was found	1-3
**Introduction**			
Background/rationale	2	Explain the scientific background and rationale for the investigation being reported	3-6
Objectives	3	State specific objectives, including any prespecified hypotheses	5,6
**Methods**			
Study design	4	Present key elements of study design early in the paper	6
Setting	5	Describe the setting, locations, and relevant dates, including periods of recruitment, exposure, follow-up, and data collection	6
Participants	6	(*a*) Give the eligibility criteria, and the sources and methods of selection of participants	6
Variables	7	Clearly define all outcomes, exposures, predictors, potential confounders, and effect modifiers. Give diagnostic criteria, if applicable	
Data sources/ measurement	8*	For each variable of interest, give sources of data and details of methods of assessment (measurement). Describe comparability of assessment methods if there is more than one group	
Bias	9	Describe any efforts to address potential sources of bias	
Study size	10	Explain how the study size was arrived at	6
Quantitative variables	11	Explain how quantitative variables were handled in the analyses. If applicable, describe which groupings were chosen and why	7,8
Statistical methods	12	(*a*) Describe all statistical methods, including those used to control for confounding	7,8
		(*b*) Describe any methods used to examine subgroups and interactions	
		(*c*) Explain how missing data were addressed	
		(*d*) If applicable, describe analytical methods taking account of sampling strategy	
		(*e*) Describe any sensitivity analyses	
**Results**			

For peer review only

Participants	13*	(a) Report numbers of individuals at each stage of study—eg numbers potentially eligible, examined for eligibility, confirmed eligible, included in the study, completing follow-up, and analysed	8
		(b) Give reasons for non-participation at each stage	
		(c) Consider use of a flow diagram	
Descriptive data	14*	(a) Give characteristics of study participants (eg demographic, clinical, social) and information on exposures and potential confounders	8
		(b) Indicate number of participants with missing data for each variable of interest	
Outcome data	15*	Report numbers of outcome events or summary measures	8-14
Main results	16	(*a*) Give unadjusted estimates and, if applicable, confounder-adjusted estimates and their precision (eg, 95% confidence interval). Make clear which confounders were adjusted for and why they were included	
		(*b*) Report category boundaries when continuous variables were categorized	
		(*c*) If relevant, consider translating estimates of relative risk into absolute risk for a meaningful time period	
Other analyses	17	Report other analyses done—eg analyses of subgroups and interactions, and sensitivity analyses	
**Discussion**			
Key results	18	Summarise key results with reference to study objectives	15
Limitations	19	Discuss limitations of the study, taking into account sources of potential bias or imprecision. Discuss both direction and magnitude of any potential bias	15
Interpretation	20	Give a cautious overall interpretation of results considering objectives, limitations, multiplicity of analyses, results from similar studies, and other relevant evidence	15-19
Generalisability	21	Discuss the generalisability (external validity) of the study results	16-19
**Other information**			
Funding	22	Give the source of funding and the role of the funders for the present study and, if applicable, for the original study on which the present article is based	20

*Give information separately for cases and controls in case-control studies and, if applicable, for exposed and unexposed groups in cohort and cross-sectional studies.

Note: An Explanation and Elaboration article discusses each checklist item and gives methodological background and published examples of transparent reporting. The STROBE checklist is best used in conjunction with this article (freely available on the Web sites of PLoS Medicine at http://www.plosmedicine.org/, Annals of Internal Medicine at http://www.annals.org/, and Epidemiology at http://www.epidem.com/). Information on the STROBE Initiative is available at www.strobe-statement.org.

For peer review only

BMJ Open

Investigating the Characteristics and Needs of Frequently Admitting Hospital Patients- A Cross-Sectional Study in the United Kingdom

Journal:	*BMJ Open*
Manuscript ID	bmjopen-2019-035522.R2
Article Type:	Original research
Date Submitted by the Author:	13-May-2020
Complete List of Authors:	Kayyali, Reem; Kingston University Faculty of Science Engineering and Computing, Pharmacy<br>Funnell, Gill; Kingston University, Kingston University Faculty of Science Engineering and Computing<br>Odeh, Bassel; Kingston University Faculty of Science Engineering and Computing, Pharmacy<br>Sharma, Anuj; exus, Exus Innovations Tower 42, 25, Old Broad St, London EC2N 1PB<br>Katsaros, Yannis; exus, Exus Innovations, Tower 42, 25, Old Broad St, London EC2N 1PB<br>Nabhani-Gebara, Shereen; Kingston University Faculty of Science Engineering and Computing, Pharmacy<br>Pierscionek, Barbara; Staffordshire University, School of Life Sciences and Education<br>Wells, Joshua; Kingston University Faculty of Science Engineering and Computing, Pharmacy<br>Chang, John; Croydon University Hospital, Chest Clinic and Research and Development
**Primary Subject Heading**:	Health informatics
Secondary Subject Heading:	Health services research
Keywords:	Quality in health care < HEALTH SERVICES ADMINISTRATION & MANAGEMENT, HEALTH SERVICES ADMINISTRATION & MANAGEMENT, Organisation of health services < HEALTH SERVICES ADMINISTRATION & MANAGEMENT

SCHOLARONE™
Manuscripts

I, the Submitting Author has the right to grant and does grant on behalf of all authors of the Work (as defined in the below author licence), an exclusive licence and/or a non-exclusive licence for contributions from authors who are: i) UK Crown employees; ii) where BMJ has agreed a CC-BY licence shall apply, and/or iii) in accordance with the terms applicable for US Federal Government officers or employees acting as part of their official duties; on a worldwide, perpetual, irrevocable, royalty-free basis to BMJ Publishing Group Ltd ("BMJ") its licensees and where the relevant Journal is co-owned by BMJ to the co-owners of the Journal, to publish the Work in this journal and any other BMJ products and to exploit all rights, as set out in our licence.

The Submitting Author accepts and understands that any supply made under these terms is made by BMJ to the Submitting Author unless you are acting as an employee on behalf of your employer or a postgraduate student of an affiliated institution which is paying any applicable article publishing charge ("APC") for Open Access articles. Where the Submitting Author wishes to make the Work available on an Open Access basis (and intends to pay the relevant APC), the terms of reuse of such Open Access shall be governed by a Creative Commons licence – details of these licences and which Creative Commons licence will apply to this Work are set out in our licence referred to above.

Other than as permitted in any relevant BMJ Author's Self Archiving Policies, I confirm this Work has not been accepted for publication elsewhere, is not being considered for publication elsewhere and does not duplicate material already published. I confirm all authors consent to publication of this Work and authorise the granting of this licence.

For peer review only

Investigating the Characteristics and Needs of Frequently Admitting Hospital Patients- A Cross-Sectional Study in the United Kingdom

Author and Co-authors:

Reem Kayyali PhD, Gill Funnell MPharm, Bassel Odeh PhD, Anuj Sharma MSc, Yannis Katsaros PhD, Shereen Nabhani-Gebara PharmD, Barbara Pierscionek PhD, Joshua Wells MPharm, John Chang FRCP

Corresponding author:

Prof Reem Kayyali

School of Life Sciences, Pharmacy and Chemistry, Kingston University,

Penrhyn Road, Kingston upon Thames

Surrey, KT1 2EE, United Kingdom

Email: R.Kayyali@kingston.ac.uk

Telephone: +44 (0)20 8417 2651 (Internal: 62561)

Co-author details:

Gill Funnell, Bassel Odeh, Shereen Nabhani-Gebara, Joshua Wells

Affiliated to

School of Life Sciences, Pharmacy and Chemistry,

Kingston University, Penrhyn Road, Kingston upon Thames

Surrey, KT1 2EE, United Kingdom

Yannis Katsaros

Affiliated to

Medidata, 12 Hammersmith Grove, London W6 7AP, United Kingdom

Anuj Sharma

Affiliated to

Exus Innovations Tower 42, 25 Old Broad Street, London EC2N 1PB, United Kingdom

John Chang

Affiliated to

Department of Research and Development, Croydon University Hospital, London Rd CR7 7YE United Kingdom

London Road CR7 7YE United Kingdom

Barbara Pierscionek

School of Life Sciences and Education, Staffordshire University, College Road, ST4 2DE

Keywords: Patient Readmission, Patient Discharge, Health Services for the Elderly, Integrated Patient Care

For peer review only

For peer review only - http://bmjopen.bmj.com/site/about/guidelines.xhtml

Word Count: 4562

ABSTRACT

OBJECTIVES: This study forms the user requirements phase of the OPTIMAL project, which, through a predictive model and supportive intervention, aims to decrease early hospital readmissions. This phase aims to investigate the needs and characteristics of patients who had been admitted to hospital ≥2 times in the past 12 months.

SETTING: This was a cross-sectional study involving patients from Croydon University Hospital (CUH), London, UK

PARTICIPANTS: A total of 347 patients responded to a postal questionnaire, a response rate of 12.7%. To meet the inclusion criteria, participants needed to be aged ≥ 18 and have been admitted ≥2 times in the previous 12 months (August 2014-July 2015) to CUH.

PRIMARY AND SECONDARY OUTCOMES: To profile patients identified as frequent admitters to assess gaps in care at discharge or post-discharge. Additionally, to understand the patients' experience of admission, discharge and post-discharge care.

RESULTS: The range of admissions in the past 12 months was 2-30, with a mean of 2.8. At discharge 72.4%, (n= 231/347) were not given a contact for out of hours help. Regression analysis identified patient factors that were significantly associated with frequent admissions (>2 in 12 months), which included age (p=0.008), being in receipt of care (p=0.005) and admission due to a fall (p=0.01), but not receiving polypharmacy. Post-discharge, 41.8% (n=145/347) were concerned about being

For peer review only - http://bmjopen.bmj.com/site/about/guidelines.xhtml

readmitted to the hospital. In the first 30 days after discharge, over half of patients (54.5% n=189/347) had no contact from a health care professional.

CONCLUSION: Considering that social care needs were more of a determinant of admission risk than medical needs, rectifying the lack of integration, communication and the under-utilisation of existing patient services could prevent avoidable problems during the transition of care and help decrease the likelihood of hospital readmission.

STRENGTHS AND LIMITATIONS OF THIS STUDY

1. The study evaluated the patient journey from admission, discharge to post-discharge, providing a holistic picture of patients' experiences
2. The study successfully implemented a cross-sectional questionnaire across a diverse sample population using a postal survey method with no reminders sent
3. The target sample included all patients ≥18 years of age who experienced ≥2 admissions in the past 12 months at CUH
4. The study utilised linear regression analysis to identify significant contributing factors to patients being admitted >2 times in a 12 month period
5. The study is representative of patients admitted only to CUH and is limited by the memory of the respondents

Introduction

A desire to reduce the increasing cost of healthcare provision is an impetus for many countries to search for new ways to both increase efficiency and improve the quality of hospital care. Reducing

For peer review only - http://bmjopen.bmj.com/site/about/guidelines.xhtml

the cost of early hospital readmissions is an objective with clear benefits for both providers and patients.[1]

In the UK, readmissions were estimated to cost the NHS £2.4 billion in 2012-2013, which is 19% of the total emergency admission cost of £12.5 billion.[2] Since 2011, UK hospitals have been financially penalised for patient readmissions occurring within 30 days of discharge, which is considered as early readmission.[3]

The UK financial penalty was introduced in 2011 to discourage hospitals from attempting to free up beds by discharging patients before they were ready.[3] However, not all early readmissions are due to sub-optimal patient care and many readmissions may be unavoidable and appropriate, for example where patients are chronically or terminally ill.[4][5] Two UK studies found around 60% of early readmissions were due to the same reason as the primary admission, suggesting that these could have been reduced by medication reviews, better discharge communication and a rapid response to preventable issues.[6][7]

Both polypharmacy and chronic conditions such as chronic obstructive pulmonary disease (COPD), cardiovascular disease (CVD) and diabetes have been found to be associated with readmission rates and increased needs following discharge.[8][9] Accurately identifying patients as high risk enables resources to be channelled specifically to these patients through supportive interventions, rather than providing for all patients, many of whom may not be at risk of readmission. Several predictive models have been developed in the UK such as PARR-30[10] and in Canada the LACE[11] with relatively good predictive accuracy.

Evaluating the effectiveness of interventions designed to prevent early readmissions is problematic due to the lack of robust studies with good methodologies.[9] Intervention types which have been studied, often in combination include: extensive discharge planning, telephone calls, home visits, a 24-hour hot line and patient education.[9] The provision of follow up telephone calls is a common

For peer review only - http://bmjopen.bmj.com/site/about/guidelines.xhtml

intervention, with variation in the number and length of calls and profession of caller. The most successful results included both pre and post-discharge interventions.[12]

Schemes for supporting patients with their medications in the community were introduced into community pharmacies in 2005. Medicine Use Reviews (MURs) and New Medicines Service (NMS) can support patients with medication adherence as well as identifying interactions and other problems. The NMS is specifically targeted at patients with long term conditions such as COPD to support patients starting a new medication.[13]

The need for successful management of the pre and post-discharge period is highlighted in the National Institute for health and Care Excellence (NICE) Guidelines[14], developed in 2015 to help with the transition of adult patients with social care needs from hospital to the community. These guidelines emphasise the importance of the transition of care being co-ordinated using good communication. All healthcare professionals (HCPs) involved with the care of the patient in hospital and the community, should be included in the communication loop, with all patients/carers being provided with a medication list and a care plan with a single HCP responsible for co-ordinating the discharge for both social and medical needs.

This paper reports on the first stage of the OPTIMAL project[15], funded by Innovate UK. The OPTIMAL project encompasses the development of a predictive risk model, together with a supportive post-discharge patient intervention with the aim of reducing early hospital readmission. Although the success of both predictive risk models and interventions to prevent hospital readmission have been developed and studied separately before, this is the first time, to our knowledge, that a predictive model and a preventative intervention have been integrated to support patients.

The aim of this study was to undertake a needs assessment to investigate any common characteristics of patients admitted more than one time to CUH in a period of 12 months and understand their experiences of both the discharge process and the immediate post-discharge

For peer review only - http://bmjopen.bmj.com/site/about/guidelines.xhtml

period. The study also sought to determine factors contributing to frequent admission (>2 in 12 months). This will assist in the development of an appropriate post-discharge intervention for patients identified at high risk of readmission.

METHODS

A cross-sectional study was carried out at Croydon University Hospital (CUH). Patients were considered for inclusion in the study if they met the following criteria: ≥18 years, a home address on the CUH database, experienced ≥ 2 admissions to CUH in the past 12 months (August 2014- July 2015). Paediatric, oncology and maternity patients were excluded from the study. CUH research and development (R&D) department using patient records identified a total of 2722 patients who met the inclusion criteria. To provide a confidence level of 95% and a confidence interval of 5%, the sample size was calculated as 337 patients. As a low response rate may be expected from postal survey, all 2722 patients were invited to complete the postal questionnaire (Supplementary File). An explanatory letter was sent with the questionnaire together with a pre-paid return envelope. The questionnaire was only made available in English and no reminders were sent.

Ethical approval was obtained from Kingston University Delegated Research Ethics Committee (Ref: 1415/035) and approved by the R&D department by CUH as a service evaluation.

A quantitative cross-sectional questionnaire survey was designed using a mixture of open and closed questions. The validated tools AUDIT-C (a brief alcohol screening tool used to identify alcohol dependency)[16] and a medical health literacy score[17] were incorporated together with other questions which investigated patient experience and knowledge of medication and discharge counselling. The questionnaire was in four sections: Firstly, demographic information, collecting personal information such as age, as well as medication list and current medical conditions. Secondly, understanding the patient's admission experience, the reason for the patient's attendance at A&E and satisfaction with the admission process. Thirdly, the patient's discharge experience,

For peer review only

investigating patients' involvement in their discharge planning and the provision of medication counselling. Finally, understanding the patients' post-discharge experience, the discharge support received by patients, as well as patients' confidence in managing their health and coping at home post-discharge. The experience sought was based on the patient's most recent admission.

Pilot

After receiving ethics approval, a pilot study was conducted which involved asking 10 patients from the discharge lounge at CUH to complete the survey for validation. Minor changes were made to the questionnaire. To prevent any bias, the findings from the pilot were not included in the final results.

Patient and Public Involvement

The study was a follow up study from 50 patients at the Trust who indicated mixed experience in counselling and shared decision making during admission. As part of the funding, the researchers agreed to inform patients/public of the outcome of the study. This was completed via the public engagement forums within the Trust.

Data Analysis

The responses from the returned questionnaires were analysed using IBM SPSS ver. 23® through descriptive statistics and the Chi-squared test for independence, with a level of significance set at 5% ($p<0.05$). A comorbidity polypharmacy score (CPS) was calculated (defined as the total of the number of pre-trauma comorbidities and the number of pre-admission medications in trauma patients ≥45 years). Our modified calculation was performed for all patients ≥45 years, using the number of medications specified in the questionnaire, together with the number of existing comorbidities recorded. A three question Audit-C score[16] was calculated, with each question having a possible score of 0-4 and giving a total score in the range between 0-12. A score of ≥ 5 is considered positive, indicating a higher risk of alcohol consumption. A single question health literacy tool was utilised giving scores of 1-5, with scores >2 indicating some difficulty reading printed health

For peer review only - http://bmjopen.bmj.com/site/about/guidelines.xhtml

Parameter	Number (n,%)	Mean (SD)	Range	Mode

material.[17] The number of medications most associated with adverse drug reactions (ADR) resulting in hospital admission was also recorded for each patient.

A linear regression analysis was carried out on the data to help identify significant patient characteristics which may have contributed to a greater number of admissions in the previous 12 months. This was carried out by adding a dependent variable column "frequent_admitter" to the data which was then assigned 1 if a patient's admissions in the previous year were >2 or 0 if ≤2. The independent variables included in the regression analysis were: admission reason, ethnicity, condition complexity indicator (which was set if a patient described their existing situation as complex/complicated or reported ≥ 2 conditions), a care indicator (identified by patients who were in receipt of some home care), CPS, patient age, gender and number of medications. The linear regression was repeated to understand the contributors to very frequent admissions, which was >3 in the previous 12 months and for those over 55 years of age with >2 admissions in the previous 12 months. Any row where any of these variables were missing was excluded, thus leaving 169 patients to be included in the regression analysis This number was 137 patients when only including those greater than 55 years of age in the regression analysis.

RESULTS

The questionnaires were sent to 2722 patients, 347 were completed and returned giving a response rate of 12.7%.

The most common reasons given for the last admission were respiratory problems such as asthma and COPD (15.0%, n=52). Nearly 10% (n=33) of patients were admitted due to a fall. Nearly a third (n=101) of patients reported more than one condition or described their condition as complex (Table 1).

Table 1 *Demographics and Medical Conditions of responders*

For peer review only - http://bmjopen.bmj.com/site/about/guidelines.xhtml

**Age (n=334)**	334, 100.0%	69.2 (18.2)	18-100	84
**Gender (n=337)**				
Male	155, 46.0%			
Female	182, 54.0%			
**Ethnicity (n=333)**				
White	250, 75.1%			
Black	34, 10.2%			
Asian	25, 7.5%			
Other	24, 7.2%			
**Medical History**				
No. of admission in previous 12 months*[1]	347, 100.0%	2.8 (1.9)	2-30	2
No. of admission in previous 30 days*[1]	32, 10.8%	1.4 (0.9)	0-6	1
**Most Common Reason for Last Admission (n=347)**				
Respiratory Conditions	52, 15.0%			
Chest Pain	18, 5.2%			
Other Pain	20, 5.8%			
Fall	33, 9.5%			
Infections excl. Chest	28, 8.1%			
Cardiac Conditions	23, 6.2%			
Other	132, 38.0%			
Not Specified	41, 11.8%			
**Most Common Existing Medical Conditions (n=347)**				
Cardiac Conditions	59, 17.0%			
Respiratory Conditions	52, 15.0%			
Hypertension	41, 11.8%			
Diabetes	42, 12.1%			
None Specified	123, 35.4%			
>1 Long Term Condition (LTC) or Described as Complex	101, 29.1%			

*[1] Number of patients admitted within previous 12 months and 30 days. Mean (SD), Range and Mode reflect number of admissions per patient sample.

For peer review only - http://bmjopen.bmj.com/site/about/guidelines.xhtml

For peer review only

Over a quarter (28.8%, n=99/344) of patients lived alone and less than 5% (4.4% n=15/344) lived in a care home. Not all patients had someone to care for them; 26.7% (n=88/330) reported that they had no available care. Only 13.1% (n=43/328) of patients currently smoked, which is less than the UK average of 19%.[18] However, 39.3 % (n=129/328) described themselves as ex-smokers. Nearly a third of patients had a limited health literacy score (29.8%, n=101/339) and over 15% (16.6%, 30/180) had a positive AUDIT-C score associated with a higher alcohol consumption risk.

Admission

Over half of patients were referred to A&E by an HCP (58.8%, n=204/347), with just over a third (34.6%, n=120/347) of patients reporting that a family member or they themselves made the decision. Although, two-thirds of patients (67.4%, n=234/347) were consulted regarding admission and care decisions, most patients (89.6%, n=311/347) wanted to be more involved with these decisions. The most frequently expressed comments about the admission experience concerned communication problems and the lack of provision of information (41.1%, n=35/85).

Regression Analysis

Five variables were found to be significantly associated with >2 admissions in the previous 12 months. These were admission for a fall (p=0.01), not identifying as having a complex condition or reporting <2 conditions (p=0.003), age (p=0.008), male gender (p=0.007) and being in receipt of care at home (p=0.005). Additionally, the overall regression is significant according to the F test (F=0.04). These factors were still significant for the sample when analysing only those patients ≥55years of age (F=0.007). The only change was that admission due to infection became significant in this sample (p=0.002). For patients with admissions >3 in 12 months CPS was found to be an additionally significant factor (p=0.02). All other independent variables were not found to have a statistically significant contribution to the frequency of admission.

Discharge

For peer review only - http://bmjopen.bmj.com/site/about/guidelines.xhtml

Nearly half of patients, (42.1%, n=146/347) were not informed of the discharge decision 24 hours in advance, including 43.4% (n=43/99) of those who lived alone.

Over half of patients, (54.0%, n=187/347) were discharged from the hospital on a weekday between 12 noon and 6pm. However, about a quarter of patients (21.3%, n=74/347) were discharged between 6pm-6am with 17.6% (n=13/74) of them living alone with an average age of 71.2 years.

Two thirds (67.4% n=234/347) of patients agreed that the decisions regarding the discharge procedure were clearly explained (Table 2). However, only a third of patients (34.3%, n=119/347) were provided with information to enable them to detect signs of deteriorating health. Furthermore, only a third of patients (33.4% n=116/347) were provided with contacts for out-of-hours support. Less than a third of patients were referred to a post-discharge service and less than half of respondents reported joining this service (Table 2).

Table 2 *Patients' Discharge Experience*

Patient Discharge Experience (n=347)	Number (n,%)
Received discharge information from a doctor	188, 54.2%
Felt the decisions at discharge were clearly explained	234, 67.4%
Was fully consulted in the decision of being discharged	226, 65.1%
Received a written copy of care plan	146, 42.1%
Told about signs or signals to watch out indicating health was worsening	119, 34.3%
Told who to contact if health deteriorated	84, 24.2%
Told who to contact for out of hours help	116, 33.4%
Referred to a post-discharge service	95, 27.4%
Patient joined the post-discharge service (**n=95**)	46, 48.4%
Provided with details of local support groups	63, 18.2%

For peer review only - http://bmjopen.bmj.com/site/about/guidelines.xhtml

When patients were asked their opinion about their discharge procedure, 72 patients responded. The main concerns expressed were the poor provision of information and communication difficulties at all levels. Patients' concerns included the lack of communication between hospital staff and the patients/patients' families (48.6% n=35/72), including two elderly patients discharged without informing their families. One patient stated: "more co-ordination is needed between the pharmacy and wards." Patients were also concerned about long waiting times (36.1% n=26/72), with 42.3% (n=11/26) of the waiting times involving a delay in receiving medications.

Medications

Two-thirds of patients reported taking at least one regular medication (67.4%, n=234/347). Three-quarters of these patients experienced changes to their medications whilst in hospital (75.2%, n=176/234), but over a quarter of these patients (28.4%, n=50/176) did not receive any counselling. Over two-thirds of patients (70.5%, n=165/234) agreed that medication information was explained in a way they could understand. However, 34.6% (n=81/234) would have liked more information regarding their medications.

The average number of medications per patient was 4.2 with of a range of 0 to 25. Nearly two-thirds (65.0%, n=152/234) of patients were taking $\geq$ 5 medications. The most commonly prescribed medication classes are shown in Table 3.

For peer review only - http://bmjopen.bmj.com/site/about/guidelines.xhtml

Table 3 *Most Common Medication Classes*

Medication Class (n=234)	Number (n,%)
Proton Pump Inhibitors	107, 45.3%
Statins	105, 44.5%
Antiplatelet drug	84, 35.6%
ACEI/ARBs (Angiotensin Converting Enzyme Inhibitors/ Angiotensin Receptor Blockers)	80, 33.9%
Beta Blockers	80, 33.9%
Calcium Channel Blockers	65, 27.5%
Loop Diuretics	52, 22.0%
Opioid analgesics (Including tramadol)	32, 13.6%
Oral Anti-Coagulants	34, 14.4%
B-2 Agonists	35, 14.8%

Some of the medication combinations found are not routinely recommended, due to being identified as risky.[19] For example, 10.7% (n=25/234) of patients were taking the high-risk combination of two or more anti-platelet drugs or an antiplatelet drug together with the anti-coagulant warfarin. Also 4.3% (n=10/234) were taking the high-risk triple combination of an (Angiotensin Converting Enzyme Inhibitors (ACEI)/ Angiotensin Receptor Blockers (ARB)), a Non-Steroidal Anti-Inflammatory Drug (NSAID) and a diuretic.

Over half of patients (56.4%, n=132/234) were prescribed two or more 2 medications that could put them at high risk of admission due to an ADR (Table 4).[20]

Table 4 *High Risk Drugs*

Number of High-Risk Meds (n=234)	Number (n,%)
>5	2, 0.9%
5	15, 6.4%
4	14, 5.9%
3	49, 20.8%
2	52, 22.0%
1	52, 22.0%
0	50, 22.0%

For peer review only

The average calculated CPS score was 7.5. Scores greater than 7 are associated with an increased risk of falls and length of hospital stay, complications, short term and one year mortality [21], over 40% of patients (n=135/313) had scores >7 and 20 patients were considered as severe or morbid with scores between 15 and 32.

Post-Discharge Experience

While 70.3% (n=244/347) of patients were confident in managing their own health, 41.8% (n=145/347) had concerns about being readmitted to the hospital, with two patients feeling that their last admission was due to medicine errors that could have been avoided. Receiving medication counselling in hospital (55.1%, n=191/347) was significantly associated with patients feeling more confident in the management of their health care issues (p=0.013). Three-quarters of patients (74.9% n=260/347), were confident in managing their supply of medicines, but were less confident in managing their social care issues (34.3%, n=119/347) and healthcare issues (48.9%, n=170/347).

Almost half of patients (46.9% n=163/347) were very satisfied or satisfied with the available support post-discharge in managing their health needs. However, less than a third of patients were satisfied (27.4% n=95/347) with the support for their social care needs.

During the crucial first 30 days post-discharge from hospital, over half (54.5%, n=189/347) of patients did not receive any contact from a hospital, GP, pharmacy, or other post-discharge service. Only 17.6%, (n=61/347) of patients reported being contacted by their GP. During this time, patients were also very reticent to contact an HCP themselves, with only 12.1% (n=42/347) of patients reporting initiating contact.

Just under a quarter of patients (24.2% n=84/347) were contacted by other post-discharge services, of the 58 patients who specified a service, half were contacted by community or other nurse, but only 15% (n=3/20) of patients suffering from COPD, 13 of which were admitted with a respiratory

For peer review only - http://bmjopen.bmj.com/site/about/guidelines.xhtml

For peer review only

problem/exacerbation, were referred to the respiratory HOT clinic (a rapid access clinic to help patients with COPD avoid hospitalisation).[22]

Community pharmacy support services were not well utilised post-discharge and only 4.0%, (n=17/347) of patients were referred to MUR, with 69.7% (n=242/347) of patients being unaware of MUR services. However, 50.4% (n=175/347) of patients were interested in receiving this service. Similarly, 78.9% (n=274/347) of patients were not referred to NMS, with 51.6% (n=179/347) of patients being interested in receiving this service.

DISCUSSION

This questionnaire-based study followed patients that had ≥2 hospital admissions/year living in the vicinity of CUH from admission, through discharge to post-discharge. Despite the low response rate, this is the first study that captures the complete patient journey from admission, discharge, through to post-discharge care. Furthermore, it identified characteristics of patients with high admission rates. A strength of this is the holistic nature of the reported data, which provides a comprehensive picture of these patients' experience of the support they were given, their physical health and medication when discharged from hospital. The data highlights a wide range of areas for improving patient support, including communication, utilisation and integration of services and medication counselling.

The study had several limitations: Firstly, it is representative of the population around CUH and admissions to that Trust only, as well as being limited by the memory of the respondents. Secondly, not all patients fully completed the questionnaire, hence, statistical significance was not achieved for the whole questionnaire. Thirdly, as the questionnaire was only available in English, this limited the study to participants who had sufficient English, the Black population was also under represented at 10.2% compared to the 2011 census figure of 20.2%.[23] Three quarters (75.1%) of patients described themselves ethnically as White, which is an over representation when compared to the Croydon borough 2011 census figure of 47.3%.[23]

For peer review only - http://bmjopen.bmj.com/site/about/guidelines.xhtml

Regression analysis identified five patient characteristics associated with higher admission. It is interesting that two of these factors: falls and being in receipt of care, both require liaison with other services post-discharge to provide adequate support in the patient's home. Suffering from falls is a well-known cause of hospital admission and corroborates with other studies[24][25], but being in receipt of care is, as far as we are aware a novel, though not surprising reason for admission. The male gender has previously been associated with increased admission, specifically in older people, which is pertinent to our study given the mean age of 69.2 years among participants.[26] Falling was the second most common reason for admission as reported by nearly 10% of patients. Polypharmacy, higher CPS score and identifying one's condition as complex or having >2 existing conditions were not significantly associated with >2 admissions in 12 months. However, a higher CPS score was found to be a significant contributor to high levels of admission (>3 in 12 months). Medications may often be implicated in falls with an increased risk for patients even those taking < 5 medications, however the medication class may be deemed to be more significant than the number.[27][28] Nevertheless, a higher CPS has been associated with an increase in falls by other studies, which may explain why this study found this factor to be significant for those that had >3 admissions in 12 months.[29][30] Nearly 50% of patients had a CPS score ≥ 7 and 65% were taking 5 or more medications. An Australian study observed a median increase from 3 to 6 annual attendances in the emergency department (ED) for those ≥65 years old who presented with comorbidities and polypharmacy (≥5 medications), among other factors.[29]

There is additional evidence to suggest that co-morbidities are a significant factor when predicting early readmission. The Charlson Index, which predicts 10-year mortality based on patients' comorbidities, was found to be significantly associated with readmission within 28 days for patients scoring ≥3 in a retrospective observational study by Li et al.[30] Interestingly, Considine et al[31] found that comorbidities were not significant predictors of readmission ≤1 day post-discharge for patients from acute-care, however health service use was notable in the 6-months preceding the index admission with ≥1 ED attendance or ≥1 hospital admission in 42.6% (n=579) and 40.7% (n=553)

For peer review only - http://bmjopen.bmj.com/site/about/guidelines.xhtml

respectively. Although our study focused primarily on frequent admission as opposed to readmission, the latter study could provide an explanation of why co-morbidities were only a predictor of high admission rate (>3 in 12 months).[31]

It must be noted that in this study, medications and conditions were self-reported. However, these were not found to be significantly associated with frequent admission (>2 in 12 months), thus highlighting that social care needs are superseding medical needs in determining increased admission risk with medical needs becoming significant in those with >3 admissions in 12 months.

Receipt of medication counselling was significantly associated with patient confidence in managing health (p=0.013). Medicine combinations were reported which could have been questioned, such as patients taking two anti-platelet drugs or an anti-platelet drug with warfarin, which can lead to an increase risk of bleeding.[19] Ten patients were taking the combination of NSAID, ACEI/ARB together with a diuretic, this combination is associated with an increased risk of acute kidney injury.[32] Community pharmacists being the most accessible HCP, are well placed to identify medications which cause adverse events to patients and increase their risk of falls. Patients were not referred to and had a lack of awareness of community pharmacy medicine information schemes - MUR or NMS. This was a missed opportunity for medication support post-discharge in the community. In fact, an initiative at CUH that piloted the provision of domiciliary MUR to housebound 'high risk' patients by community pharmacists resulted in reported avoidance of hospitalisation.[33][34]

Although nearly three quarters of patients felt consulted in the decisions leading to their discharge, patients expressed dissatisfaction with the discharge process, with long waiting times, delays and poor communication reported as the most common complaints. These findings correlate with an AGE UK report[35] investigating older people's experience of hospital readmission. Delays in discharge and lack of information are upsetting and confusing. Patients should at least be provided with updates as to the progress of their discharge. Although this study is limited to the experiences

For peer review only - http://bmjopen.bmj.com/site/about/guidelines.xhtml

of the population around Croydon, a study from Liverpool Hospital UK[36] reported similar percentages of patients (70%) who felt that discharge decisions were explained, with the long wait for discharge medications also having a negative influence on the discharge experience.

Nearly 50% of patients were worried about being readmitted to hospital and commented on finding the experience stressful and wanting to avoid readmission. Good communication and information sharing supports the transition from hospital and helps prevent readmission.[14][37] Contact information should be provided in case of a short-term crisis, which should be proactive rather than waiting for a more serious problem to arise. However, it was found that nearly 40% of patients were not provided with the signs of deterioration of their condition and nearly three quarters of patients were not provided with details of who to contact if this situation arose. This lack of information could result in patients returning to hospital. Additionally, patients' carers and families were not always informed of the discharge, making it hard for them to adequately support the patient at home.

Poor integration of services was found both within the hospital and between primary and secondary care providers. Patients with social care needs should be contacted by a GP or community nurse within 24-72 hours of discharge.[14] However, less than 20% of patients were contacted by their GP within 30 days of discharge. A further 12.1% contacted a HCP themselves. Additionally, patients were not being referred to post-discharge services which could have supported them. Despite 20 patients reporting suffering from COPD and 13 of these patients reporting respiratory problems/exacerbation as the reason for admission, only 3 patients were referred to the respiratory HOT clinic at CUH[22] which provides an integrated team of multidisciplinary HCPs. Nearly a third of patients were dissatisfied with their social care, thus it is not surprising that those receiving care were more at risk of frequent admission. A lack of transition of care was reported, with a need for low level practical support during the first few days after discharge. This is a shared outcome with the AGE UK report.[35]

For peer review only - http://bmjopen.bmj.com/site/about/guidelines.xhtml

More integrated support such as that provided by Lewisham Integrated Medicines Optimisation Scheme (LIMOS)[38] can break through traditional boundaries of care, but as these authors note such links with services take time to build. With an increasing aging population with more multi-morbidities, the integration of service delivery across different clinical areas becomes more important to provide appropriate individual care, rather than the current disease-focused practice.[39] A move to a shared responsibility, is required across multiple areas- social, voluntary and clinical, to provide the integrated personalised care that patients need.[40]

CONCLUSIONS

The study highlighted gaps in care during the patient discharge journey. Admission for a fall and receipt of care were significantly associated with higher admission rates. Additionally, it reports for the first time, that social care is an important determinant of frequent admission (>2 in 12 months) in a predominantly older population. Before discharge, patients lacked medication counselling, information on symptoms of deteriorating health, or HCP to contact if this situation arose. An improvement in communications at all levels would benefit patients, ensuring patients are informed of delays and decisions. Additionally, patients' confidence in their care being well managed may be increased by demonstrating that communication channels are open between different HCPs. Post-discharge, patients were lacking referrals to relevant services which could have supported them. The study highlighted that transitional care is fragmented between different services of primary, secondary and social care as well as the voluntary sector. This lack of integration is causing patients avoidable difficulties. Improvement could be made by increasing HCP awareness of the available services, both voluntary and statutory, in the local area and encouraging links. Integrating services would increase the utilisation of existing resources, such as community pharmacy medicine support schemes, hospital services, e.g. respiratory HOT clinics as well as voluntary services, with care pathways utilising all relevant services across each sector.

For peer review only - http://bmjopen.bmj.com/site/about/guidelines.xhtml

CONTRIBUTORSHIP STATEMENT RK was the principal investigator of the study. She was responsible for the design of the study. She also organised and co-ordinated all aspects of this research. GF worked alongside RK to draft the publication. BO contributed to data collection. The analysis of the results was carried out by RK, GF, BP, SNG, YK, AS, JW and JC.

COMPETING INTERESTS All authors have completed the ICMJE form for disclosure of potential conflicts of interest available from www.icmje.org/coi_disclosure.pdf and declare that there is nothing to disclose.

FUNDING: This research was carried out as part of the OPTIMAL project which has received funding from Innovate UK.

DATA SHARING STATEMENT No additional data is available

1. OECD Health Ministerial Meeting: Health System Priorities in the Aftermath of the Crisis [Internet]. 2010 [cited 2017 Feb 7]. p. 7–8. Available from: https://www.oecd.org/health/2010-ministerial/46098360.pdf
2. National Audit Office. Emergency admissions to hospital: managing the demand [Internet]. Vol. 739, Hc. 2013 [cited 2017 Feb 10]. p. 4. Available from: https://www.nao.org.uk/wp-content/uploads/2013/10/10288-001-Emergency-admissions.pdf
3. Department of Health. Payment by Results Guidance for 2011-12 [Internet]. 2011 [cited 2017 Feb 17]. Available from: http://webarchive.nationalarchives.gov.uk/20130507170152/https://www.gov.uk/government/uploads/system/uploads/attachment_data/file/151911/dh_126157.pdf.pdf
4. Benbassat J, Taragin M. Hospital Readmissions as a Measure of Quality of Health Care. arch intern med. 2000;160:1074–81.
5. Lindquist LA, Baker DW. Understanding preventable hospital readmissions: Masqueraders, markers, and true causal factors. J Hosp Med. 2011;6(2):51–3.
6. Conroy SP et al. Understanding readmissions: An in-depth review of 50 patients readmitted back to an acute hospital within 30 days. Eur Geriatr Med [Internet]. 2013;4(1):25–7. Available from: http://dx.doi.org/10.1016/j.eurger.2012.02.007
7. Jakupaj A. Reducing readmission rate in the elderly population at a District General Hospital. Eur Geriatr Med [Internet]. 2014;5:S248–9. Available from: http://ovidsp.ovid.com/ovidweb.cgi?T=JS&PAGE=reference&D=emed12&NEWS=N&AN=71659663
8. Hurding S et al. Polypharmacy Guidance 2015 (NHS Scotland). 2015.
9. Jayakody A et al. Effectiveness of interventions utilising telephone follow up in reducing hospital readmission within 30 days for individuals with chronic disease: a systematic review.

For peer review only - http://bmjopen.bmj.com/site/about/guidelines.xhtml

BMC Health Serv Res [Internet]. 2016;16(1):403. Available from: http://www.ncbi.nlm.nih.gov/pubmed/27538884%5Cnhttp://www.pubmedcentral.nih.gov/articlerender.fcgi?artid=PMC4990979

10. Billings J et al. Development of a predictive model to identify inpatients at risk of re-admission within 30 days of discharge (PARR-30). BMJ Open [Internet]. 2012;2(4):e001667–e001667. Available from: http://www.ncbi.nlm.nih.gov/pubmed/22885591%5Cnhttp://www.pubmedcentral.nih.gov/articlerender.fcgi?artid=PMC3425907

11. Spiva L et al. Validation of a Predictive Model to Identify Patients at High Risk for Hospital Readmission. J Heal Qual. 2015;00(0):1–7.

12. Sales VL et al. Utilization of Trained Volunteers Decreases 30-Day Readmissions for Heart Failure. J Card Fail [Internet]. 2014;20(5):377–377.e23. Available from: http://linkinghub.elsevier.com/retrieve/pii/S1071916414001432

13. NHS. Medicine Use Reviews [Internet]. [cited 2017 Apr 10]. Available from: http://www.nhsemployers.org/your-workforce/primary-care-contacts/community-pharmacy/medicines-use-reviews-murs

14. NICE NG27. NICE Transition between inpatient hospital settings and community or care home settings for adults with social care needs NG27 [Internet]. 2015 [cited 2017 Mar 5]. p. 1–29. Available from: https://www.nice.org.uk/guidance/ng27

15. EXUS Innovations. OPTIMAL project [Internet]. [cited 2017 Apr 10]. Available from: https://www.exusinnovation.co.uk/en/optimal/

16. Bush K et al. The AUDIT Alcohol Consumption Questions (AUDIT-C) An Effective Brief Screening Test for Problem Drinking. Arch Intern Med. 1998;158(16):1789–95.

17. Chew LD et al. Validation of screening questions for limited health literacy in a large VA outpatient population. J Gen Intern Med. 2008;23(5):561–6.

18. Centre H and SCI. Statistics on Smoking - Key facts [Internet]. 2016 [cited 1917 Jan 9]. Available from: http://content.digital.nhs.uk/catalogue/PUB20781/stat-smok-eng-2016-app.pdf

19. All Wales Medicines Strategy Group. Polypharmacy: Guidance for Prescribing. July 2014. [Internet]. 2014 [cited 2017 Mar 7]. Available from: http://www.awmsg.org/docs/awmsg/medman/Polypharmacy - Guidance for Prescribing.pdf

20. Pirmohamed M et al. Adverse drug reactions as cause of admission to hospital: prospective analysis of 18 820 patients. Bmj. 2004;329(7456):15–9.

21. Housley BC et al. Comorbidity-polypharmacy score predicts readmission in older trauma patients. J Surg Res [Internet]. 2015;199(1):237–43. Available from: http://dx.doi.org/10.1016/j.jss.2015.05.014

22. Croydon Health Care Trust. HOT Clinics [Internet]. 2012 [cited 2017 Apr 19]. Available from: https://www.croydonhealthservices.nhs.uk/new_page.htm

23. UK Office For National Statistics. 2011 UK Census Aggregate Data [Internet]. 2011 [cited 2017 Apr 10]. Available from: https://www.ons.gov.uk/census/2011census

24. Hughes LD, Witham MD. Causes and correlates of 30 day and 180 day readmission following discharge from a Medicine for the Elderly Rehabilitation unit. BMC Geriatr. 2018;18(1):1–10.

For peer review only - http://bmjopen.bmj.com/site/about/guidelines.xhtml

25. Hallgren J, Aslan AKD. Risk factors for hospital readmission among Swedish older adults. Eur Geriatr Med [Internet]. 2018;9(5):603–11. Available from: https://doi.org/10.1007/s41999-018-0101-z

26. Cornette P et al. Differential risk factors for early and later hospital readmission of older patients. Aging clinical and experimental research. 2005 Aug 1;17(4):322-8.

27. Helgadóttir B et al. Medication and fall injury in the elderly population; Do individual demographics, health status and lifestyle matter? BMC Geriatr. 2014;14(1):1–8.

28. Fritsch MA, Shelton PS. Geriatric Polypharmacy: Pharmacist as Key Facilitator in Assessing for Falls Risk. Clin Geriatr Med. 2017;33(2):205–23.

29. Berry D, Street M, Considine J. Service use by older very frequent emergency department users: A retrospective cohort study. Australasian emergency care. 2019 Sep 1;22(3):133-8.

30. Li JY et al. Identifying risk factors and patterns for unplanned readmission to a general medical service. Australian Health Review. 2015 Feb 24;39(1):56-62.

31. Considine J et al. Factors associated with unplanned readmissions in a major Australian health service. Australian Health Review. 2019 Feb 18;43(1):1-9.

32. Lapi F et al. Concurrent use of diuretics, angiotensin converting enzyme inhibitors, and angiotensin receptor blockers with non-steroidal anti-inflammatory drugs and risk of acute kidney injury: nested case-control study. BMJ [Internet]. 2013;346(jan08_12):e8525. Available from: http://www.bmj.com/content/346/bmj.e8525

33. Jesson B, Williams V. Domiciliary Medicine Review Service in Croydon - Presentation [Internet]. Pharmacy Management National Forum - Forum Workshop. 2015 [cited 2017 Apr 7]. Available from: http://www.pharman.co.uk/imagelib/pdfs/Domiciliary_Medicine_Review_Service_in_Croydon_-_Barbara_Jesson_and_Victoria_Williams.pdf

34. Williams V et al. Domiciliary MURs: our experience in Croydon [Internet]. Pharmaceutical Journal. 2012 [cited 2017 Apr 9]. Available from: http://www.pharmaceutical-journal.com/opinion/correspondence/domiciliary-murs-our-experience-in-croydon/11109461.article

35. Lawrie M, Battye F. Older people's experience of emergency hospital readmission [Internet]. Age UK. 2012 [cited 2017 Mar 9]. Available from: http://www.ageuk.org.uk/Documents/EN-GB/For-professionals/Research/Emergency_readmission_older_peoples_experiences.pdf?dtrk=true

36. Wright S et al. UK hospital patient discharge: the patient perspective. Eur J Hosp Pharm. 2017;(January).

37. Winfield A, Burns E. Let's all get home safely : a commentary on NICE and SCIE guidelines (NG27) transition between inpatient hospital settings and community or care home settings. Age Ageing. 2016;45(August):757–60.

38. Lai K et al. Lewisham integrated medicines optimisation service: delivering a system-wide coordinated care model to support patients in the management of medicines to retain independence in their own home. Eur J Hosp Pharm. 2015;22(2):98–101.

39. Stange KC, Ferrer RL. The Paradox of Primary Care (editorial). Ann Fam Med [Internet]. 2009;7(4):293–9. Available from:

For peer review only - http://bmjopen.bmj.com/site/about/guidelines.xhtml

https://www.ncbi.nlm.nih.gov/pmc/articles/PMC2713149/pdf/0060293.pdf

40. Valentijn PP et al. Understanding integrated care: a comprehensive conceptual framework based on the integrative functions of primary care. Int J Integr Care. 2013;13(March).

For peer review only

For peer review only - http://bmjopen.bmj.com/site/about/guidelines.xhtml

Section A: YOUR ADMISSION EXPERIENCE

This part is about your experience while you were being ***admitted and treated*** *at Croydon University Hospital.*

1) What was the reason for your last admission at Croydon University Hospital?

2) Who decided that you need to go to A&E?

- ☐ Self
- ☐ Family
- ☐ GP
- ☐ Ambulance paramedic
- ☐ I don't know
- ☐ Other *(please specify)*

3) Did you try to seek help from any of the following before attending A&E? ***(please select all that apply)***

- ☐ None. I went directly to A&E
- ☐ Calling 111
- ☐ Contacting GP
- ☐ Visiting Walk-in centre
- ☐ Self-Care from the Pharmacy
- ☐ Community nurses
- ☐ HOT Clinics
- ☐ Other *(please specify)*

4) What day/time did you arrive to A&E?*(please select one box only)*

Day/Time	6am-12noon	12noon - 6pm	6pm-12midnight	12midnight - 6am
Weekday (Mon-Fri)	☐	☐	☐	☐
Weekend (Sat-Sun)	☐	☐	☐	☐

5) Following your arrival at A&E, how long did you wait before you were examined by a doctor?

- ☐ 1-2 Hours
- ☐ 2-3 Hours
- ☐ 3-4 Hours
- ☐ > 4 Hours *(please specify)*

6) Following examination by a doctor, how long did you wait before you were admitted to a bed in the ward?

- ☐ 1-2 Hours
- ☐ 2-3 Hours
- ☐ 3-4 Hours
- ☐ > 4 Hours *(please specify)*

7) To what extent do you agree/disagree that the following were explained to you in a way you could clearly understand

i The reasons for your admission

- ☐ Strongly Disagree
- ☐ Disagree
- ☐ Neutral
- ☐ Agree
- ☐ Strongly Agree

ii The decisions regarding your care, treatment, and/or procedure

- ☐ Strongly Disagree
- ☐ Disagree
- ☐ Neutral
- ☐ Agree
- ☐ Strongly Agree

8) Were you consulted regarding the decisions about your care, treatment, and/or procedure on admission?

- ☐ Yes
- ☐ No
- ☐ Can't Remember

9) In the future, would you like to be involved in decisions about your care, treatment, or procedure?

- ☐ Yes
- ☐ No
- ☐ Can't Remember

10) What could have improved your experience while being admitted to the hospital?

PROPRIETARY RIGHTS STATEMENT

This document contains information, which is proprietary to the OPTIMAL Consortium. Neither this document nor the information contained herein shall be used, duplicated or communicated by any means to any third party, in whole or in parts, except with prior written consent of the OPTIMAL consortium.

For peer review only

Section B: YOUR DISCHARGE EXPERIENCE

*The following part is about your experience while you were being **discharged from the hospital**.*

1) Were you informed 24 hours in advance about the discharge decision?

- ☐ Yes
- ☐ No
- ☐ Can't Remember

2) What day/time were you discharged from the hospital? *(Please tick one box only)*

Day/Time	6am-12noon	12noon-6pm	6pm-12midnight	12midnight-6am
Weekday (Mon-Fri)	☐	☐	☐	☐
Weekend (Sat-Sun)	☐	☐	☐	☐

3) Who provided you with the information related to your discharge? *(please select all that apply)*

- ☐ Doctor
- ☐ Nurse
- ☐ Pharmacist
- ☐ No one
- ☐ Other (please specify)

4) To what extent do you agree/disagree with the following:

i The decisions regarding my discharge were explained to me in a way I could clearly understand

- ☐ Strongly Disagree
- ☐ Disagree
- ☐ Neutral
- ☐ Agree
- ☐ Strongly Agree

ii I was fully consulted with the decision of being discharged from the hospital

- ☐ Strongly Disagree
- ☐ Disagree
- ☐ Neutral
- ☐ Agree
- ☐ Strongly Agree

iii Staff took my preferences into account in deciding how my health care will be managed when I will leave the hospital

- ☐ Strongly Disagree
- ☐ Disagree
- ☐ Neutral
- ☐ Agree
- ☐ Strongly Agree

5) Was there any change in your medicines during your last hospital admission?

- ☐ Yes
- ☐ No
- ☐ Can't Remember

6) Were you provided with any counselling about your medication(s)?

- ☐ Yes (go to 7)
- ☐ No (go to 12)
- ☐ Can't Remember

7) Who provided you with the information related to your medication(s) at discharge?
(please select all that apply)

- ☐ Doctor
- ☐ Nurse
- ☐ Pharmacist
- ☐ Other please specify

8) How was this information given to you?

- ☐ Verbally
- ☐ Written
- ☐ Verbally & Written

9) What resources were you given to help you take your medicine(s)? *(please select all that apply)*

- ☐ Patient information leaflet in box
- ☐ Medication reminder card
- ☐ Medication record book
- ☐ Poster or brochure
- ☐ None
- ☐ Other (please specify)

10) To what extent do you agree/disagree with the following:

i The information about my medication(s) were given/explained to me in a way I could clearly understand

- ☐ Strongly Disagree
- ☐ Disagree
- ☐ Neutral
- ☐ Agree
- ☐ Strongly Agree

ii I would like to have more information regarding my medicines

- ☐ Strongly Disagree
- ☐ Disagree
- ☐ Neutral
- ☐ Agree
- ☐ Strongly Agree

For peer review only - http://bmjopen.bmj.com/site/about/guidelines.xhtml

11) Please rate your satisfaction with regards to the following information about your medication, if provided during the counselling session:

Please tick one box for each row	Very Unsatisfied	Unsatisfied	Neutral	Satisfied	Very Satisfied	Not provided
Purpose of your medicine(s)	☐	☐	☐	☐	☐	☐
How to take/use the medicine(s)	☐	☐	☐	☐	☐	☐
Important side effects	☐	☐	☐	☐	☐	☐
Actions to take if you get any important side effects.	☐	☐	☐	☐	☐	☐
Lifestyle changes associated with taking your medicine(s)	☐	☐	☐	☐	☐	☐

For peer review only

For peer review only - http://bmjopen.bmj.com/site/about/guidelines.xhtml

12) Community pharmacies are offering a New Medicines Service (NMS) which is an open conversation between you and the pharmacist to discuss any concerns you may have about your new medicine(s) - for example side effects

i Are you aware of this service?
- Yes
- No

ii Were you offered/referred to this service upon discharge?
- Yes
- No
- Not Sure

iii Would you have been interested to be referred to this service?
- Yes
- No
- Not Sure

13) Community pharmacies are offering a Medicines Use Review (MUR) which is an open conversation between you and the pharmacist to discuss your medications after you were discharged from the hospital or periodically.

i Are you aware of this service?
- Yes
- No

ii Were you offered/referred to this service upon discharge?
- Yes
- No
- Not Sure

iii Would you have been interested to be referred to this service?
- Yes
- No
- Not Sure

14) When discharged from the hospital, were you given a written copy of your care plan?

☐ Yes
☐ No *(go to 15)*

i If yes, did you understand what was in this care plan?

☐ Yes
☐ No

15) **Were you referred to a post-discharge service***? (e.g. hospital avoidance team, hot clinics, telehealth, community services nurse, social care)*

☐ Yes
☐ No *(go to 16)*

i If yes, please specify

ii Were you offered a choice to select those services?

☐ Yes
☐ No

iii Have you joined any of these services yet?

☐ Yes
☐ No

16) Were you told about signs/signals of worsening or decline of your health to watch out for?

☐ Yes
☐ No *(go to 17)*

i If yes, were you given details of who to contact if this happened?

☐ Yes
☐ No

17) Were you given contacts for out-of-hours help?

☐ Yes
☐ No

For peer review only - http://bmjopen.bmj.com/site/about/guidelines.xhtml

18) **Were you given information about local support groups?** *(E.g. Diabetes UK, Age UK, Cardiac Support Group, Breathe Easy groups, etc…)*

☐ Yes
☐ No

19) What could have improved your experience while being discharged from the hospital?

For peer review only

Section C: YOUR POST-DISCHARGE EXPERIENCE

This part is about your experience since your ***last hospital discharge***

1 How confident are you in managing your health?

- ☐ Not at all confident
- ☐ Not confident
- ☐ Neither
- ☐ Confident
- ☐ Completely Confident

i Why is that?

2 How worried have you been about being readmitted to the hospital?

- ☐ Very Worried
- ☐ Worried
- ☐ Neither
- ☐ Not Worried
- ☐ Not worried at all

i Why is that?

3 Please rate your satisfaction with regards to the following

Please tick one box for each row	Very Unsatisfied	Unsatisfied	Neutral	Satisfied	Very Satisfied	Not Applicable
The available support to manage your health after you were discharged from the hospital	☐	☐	☐	☐	☐	☐
Social care support after being discharged from the hospital *(e.g. help in your home, community support, etc…)*	☐	☐	☐	☐	☐	☐
Occupational needs support *(e.g. walking aids)*	☐	☐	☐	☐	☐	☐

4 When discharged from the hospital, were you given any follow up appointments?

- Yes
- No *(please go to 5)*
- Not Sure *(please go to 5)*

i If yes, who was the follow up appointment with? *(please select all that apply)*

- GP
- Nurse
- Hospital Outpatient
- Pharmacist
- Other (please specify)

ii Were you able to attend all these appointments?

- Yes
- No

iii If no, why is that? (Optional)

iv To what extent do you agree/disagree with the following:

(a) I clearly understand my post-discharge care plan/appointments

- Strongly Disagree
- Disagree
- Neutral
- Agree
- Strongly Agree

(b) I could easily keep track/record of my appointments

- Strongly Disagree
- Disagree
- Neutral
- Agree
- Strongly Agree

(c) I prefer to have my appointments available for me electronically (e.g. smart phone calendar)

- Strongly Disagree
- Disagree
- Neutral
- Agree
- Strongly Agree

5 Have you received any follow up from the hospital?

- Yes
- No *(please go to 6)*

For peer review only

- Don't remember *(please go to 6)*

i If yes, how? *(please select all that apply)*
- By phone call
- By letter
- By email
- By text message (SMS)
- Other (please specify)

ii When?
- 1 week after discharge
- 2 week after discharge
- 3 week after discharge
- 4 week after discharge
- Other (please specify)

6 Have you been contacted by any other post-discharge service team? (e.g. hospital avoidance team, hot clinics, telehealth, community services nurse, social care)
- Yes
- No *(please go to 7)*
- Don't remember *(please go to 7)*

i If yes, who was it? (please specify)

ii How? *(please select all that apply)*
- By phone call
- By letter
- By email
- By text message (SMS)
- Other *(please specify)*

iii When?
- 1 week after discharge
- 2 week after discharge
- 3 week after discharge
- 4 week after discharge
- Other *(please specify)*

7 During the first 30 days after you were discharged from the hospital:

i Were you contacted by any of the following *(please select all that apply)*
- GP
- Hospital
- Pharmacy Team
- Post-discharge Service
- None
- Other *(please specify)*

ii Did you contact any of the following *(please select all that apply)*
- GP
- Hospital
- Pharmacy Team
- Post-discharge Service
- None
- Other *(please specify)*

8 How confident are you regarding the management of the following:

Please tick one box for each row	Not at all confident	Not confident	Neither	Confident	Completely Confident	Not applicable
Your supply of medicines	☐	☐	☐	☐	☐	☐
Your social care issues	☐	☐	☐	☐	☐	☐
Your healthcare issues	☐	☐	☐	☐	☐	☐

For peer review only - http://bmjopen.bmj.com/site/about/guidelines.xhtml

Section D: SOME INFORMATION ABOUT YOU

1) How many times were you admitted to the hospital

in the last 12 months?	
in the last 30 days?	

1) **In what year were you born?**

2) What is the first part of your postcode? (E.g. if your post code is CR7 7YE, please write CR7)

3) What is your gender?

□ **Male** □**Female**

4) How would you describe your ethnicity?

- White
- Black
- Chinese
- Mixed
- Asian
- Other
- **Prefer** not to say

5) What is/are the main language(s) spoken at your home?

6) How often do you need to have someone help you when you read instructions, pamphlets, or other written material from your doctor or pharmacy

- Never
- Rarely
- Sometimes
- Often
- Always

7) Do you suffer from any medical conditions? If yes please specify

8) How often do you have a drink containing alcohol?

- Never *(please skip to 11)*
- Monthly or less
- 2 to 4 times a MONTH
- 2 to 3 times a WEEK
- 4 or more times a week

9) How many drinks of alcohol do you drink on a typical day when you are drinking?

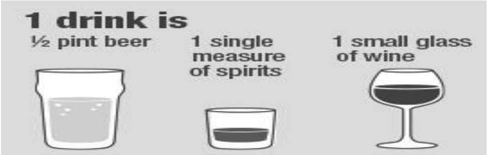

- 1 or 2 drinks
- 3 or 4 drinks
- 5 or 6 drinks
- 7 or 8 or 9 drinks
- 10 **or** more drinks

For peer review only - http://bmjopen.bmj.com/site/about/guidelines.xhtml

For peer review only

10) How often have you had 6 or more units on a single occasion in the last year?

- Never
- Less than monthly
- Monthly
- Weekly
- **Daily** or almost daily

11) Have you ever smoked a cigarette, a cigar or a pipe?

- Yes
- No *(please go to 12)*

i Yes, and I am currently a smoker

- How frequently do you smoke cigarettes?
  - Regularly
  - Occasionally
- (b) How many cigarettes do you smoke per day?
  - 10 or Less
  - 11-20
  - 21-30
  - 31 or More

ii Yes, but I am an ex-smoker

- How frequently did you smoke cigarettes?
  - Regularly
  - Occasionally
- (b) About how many cigarettes did you smoke in a day?
  - ☐ 10 or Less
  - ☐ 11-20
  - ☐ 21-30
  - ☐ 31 or More
- (c) For approximately how many years did you smoke cigarettes regularly?
- (d) How long ago did you stop smoking cigarettes?

iii Are you currently using e-cigarettes?

- ☐ Yes
- ☐ No

12) How many regular medicines are you currently taking?

13) How often do you miss doses of your medicine(s), cut down or stop taking them?

- ☐ Frequently (more than once a week)
- ☐ Occasionally (once a week)
- ☐ Rarely (once a month)
- ☐ Very rarely (once every 6 month)
- ☐ Never

14) Do you experience any of the following problems with your medicines which make it difficult for you to take your medicine(s)? (please select all that apply)

- ☐ Forget
- ☐ Cost
- ☐ Difficulty understanding the instructions
- ☐ Difficulty reading labels
- ☐ Difficulty opening containers

For peer review only

For peer review only - http://bmjopen.bmj.com/site/about/guidelines.xhtml

- ☐ Difficulty in administration *(e.g. difficulty in using Inhalers, swallowing tablets, or injection site, etc…)*
- ☐ Other *(please specify)*

15) Usually, how are your medicines supplied to you?

☐ Original or labelled box/bottle	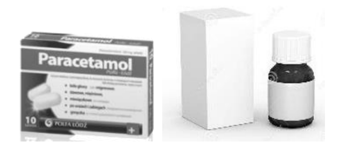
☐ Blister pack	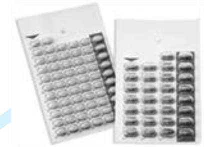
☐ Medicine Dosettes	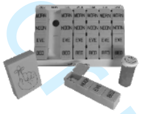

16) If known, please list your current medicines names

17) Who lives with you at home?

- ☐ I live alone
- ☐ I live with a family member
- ☐ I live with a friend
- ☐ I live in a care home
- ☐ Other please specify

18) Do you have someone who can take care of you?

- ☐ Yes
- ☐ No

i. Who provides you with home care?

- ☐ Family member
- ☐ Friend
- ☐ Carer
- ☐ Other please specify

ii. How many days a week are he/she/they available to you?

- ☐ < 1day
- ☐ 1-2 days
- ☐ 3-4 days
- ☐ 5-7 days

iii. When are they available to you?

For peer review only

- ☐ Weekdays only
- ☐ Weekend only
- ☐ Both

For peer review only

For peer review only - http://bmjopen.bmj.com/site/about/guidelines.xhtml

STROBE 2007 (v4) Statement—Checklist of items that should be included in reports of *cross-sectional studies*

Section/Topic	Item #	Recommendation	Reported on page #
**Title and abstract**	1	(*a*) Indicate the study's design with a commonly used term in the title or the abstract	1
		(*b*) Provide in the abstract an informative and balanced summary of what was done and what was found	1, 2
**Introduction**			
Background/rationale	2	Explain the scientific background and rationale for the investigation being reported	3-5
Objectives	3	State specific objectives, including any prespecified hypotheses	5
**Methods**			
Study design	4	Present key elements of study design early in the paper	6
Setting	5	Describe the setting, locations, and relevant dates, including periods of recruitment, exposure, follow-up, and data collection	6
Participants	6	(*a*) Give the eligibility criteria, and the sources and methods of selection of participants	6
Variables	7	Clearly define all outcomes, exposures, predictors, potential confounders, and effect modifiers. Give diagnostic criteria, if applicable	N/A
Data sources/ measurement	8*	For each variable of interest, give sources of data and details of methods of assessment (measurement). Describe comparability of assessment methods if there is more than one group	N/A
Bias	9	Describe any efforts to address potential sources of bias	N/A
Study size	10	Explain how the study size was arrived at	6
Quantitative variables	11	Explain how quantitative variables were handled in the analyses. If applicable, describe which groupings were chosen and why	7
Statistical methods	12	(*a*) Describe all statistical methods, including those used to control for confounding	7
		(*b*) Describe any methods used to examine subgroups and interactions	N/A
		(*c*) Explain how missing data were addressed	N/A
		(*d*) If applicable, describe analytical methods taking account of sampling strategy	N/A
		(*e*) Describe any sensitivity analyses	N/A
**Results**			

For peer review only

Participants	13*	(a) Report numbers of individuals at each stage of study—eg numbers potentially eligible, examined for eligibility, confirmed eligible, included in the study, completing follow-up, and analysed	8
		(b) Give reasons for non-participation at each stage	N/A
		(c) Consider use of a flow diagram	N/A
Descriptive data	14*	(a) Give characteristics of study participants (eg demographic, clinical, social) and information on exposures and potential confounders	8
		(b) Indicate number of participants with missing data for each variable of interest	N/A
Outcome data	15*	Report numbers of outcome events or summary measures	8-13
Main results	16	(*a*) Give unadjusted estimates and, if applicable, confounder-adjusted estimates and their precision (eg, 95% confidence interval). Make clear which confounders were adjusted for and why they were included	N/A
		(*b*) Report category boundaries when continuous variables were categorized	N/A
		(*c*) If relevant, consider translating estimates of relative risk into absolute risk for a meaningful time period	N/A
Other analyses	17	Report other analyses done—eg analyses of subgroups and interactions, and sensitivity analyses	N/A
**Discussion**			
Key results	18	Summarise key results with reference to study objectives	14
Limitations	19	Discuss limitations of the study, taking into account sources of potential bias or imprecision. Discuss both direction and magnitude of any potential bias	14
Interpretation	20	Give a cautious overall interpretation of results considering objectives, limitations, multiplicity of analyses, results from similar studies, and other relevant evidence	16-17
Generalisability	21	Discuss the generalisability (external validity) of the study results	16-17
**Other information**			
Funding	22	Give the source of funding and the role of the funders for the present study and, if applicable, for the original study on which the present article is based	18

*Give information separately for cases and controls in case-control studies and, if applicable, for exposed and unexposed groups in cohort and cross-sectional studies.

Note: An Explanation and Elaboration article discusses each checklist item and gives methodological background and published examples of transparent reporting. The STROBE checklist is best used in conjunction with this article (freely available on the Web sites of PLoS Medicine at http://www.plosmedicine.org/, Annals of Internal Medicine at http://www.annals.org/, and Epidemiology at http://www.epidem.com/). Information on the STROBE Initiative is available at www.strobe-statement.org.

For peer review only
